# Fungal-Derived Decahydrofluorene Alkaloids Promote Mitochondrial Resilience and Neuroprotection in Cellular and Animal Models of Parkinson’s Disease

**DOI:** 10.3390/antiox15091151

**Published:** 2026-09-10

**Authors:** Alberto Vázquez-Jiménez, Margarita M. Marques, José M. Sánchez, Jesús Agulla, Rebeca Lapresa, Mónica Trigal-Martínez, Rosalía Fernández-Alonso, Gracia Merino, Antonio Fernández, Antonella Consiglio, Juan P. Bolaños, Ángeles Almeida, María C. Marín, Lorena López-Ferreras

**Affiliations:** 1 Instituto de Biomedicina and Departamento de Biología Molecular, Universidad de León, 24071 León, Spain; avazj@unileon.es (A.V.-J.); rfera@unileon.es (R.F.-A.); 2 Instituto de Investigación Biosanitaria de León, 24071 León, Spain; mmarm@unileon.es; 3 Instituto de Desarrollo Ganadero y Sanidad Animal and Departamento de Producción Animal, Universidad de León, 24071 León, Spain; 4 Biomar Microbial Technologies, Parque Tecnológico de León, 24009 León, Spainmonicatrigal@gmail.com (M.T.-M.); afernandezmedarde@gmail.com (A.F.); 5 Instituto de Biología Funcional y Genómica, Universidad de Salamanca, Consejo Superior de Investigaciones Científicas (CSIC), 37007 Salamanca, Spain; jesusfreire@usal.es (J.A.); rebecalrg@usal.es (R.L.); jbolanos@usal.es (J.P.B.); aaparra@usal.es (Á.A.); 6 Instituto de Investigación Biomédica de Salamanca, Universidad de Salamanca, Consejo Superior de Investigaciones Científicas (CSIC), 37007 Salamanca, Spain; 7 Instituto de Investigación Biomédica de Salamanca (IBSAL), Hospital Universitario de Salamanca, Universidad de Salamanca, CSIC, 37007 Salamanca, Spain; 8 Instituto de Desarrollo Ganadero y Sanidad Animal, Universidad de León, 24071 León, Spain; gmerp@unileon.es; 9 Departamento de Ciencias Biomédicas, Universidad de León, 24071 León, Spain; 10 Departament de Patologia i Terapèutica Experimental, Hospital Universitario de Bellvitge-(IDIBELL), 08908 L’Hospitalet de Llobregat, Spain; consiglio@ub.edu; 11 Institut de Biomedicina, Universitat de Barcelona, 08028 Barcelona, Spain

**Keywords:** Parkinson’s disease, neuroprotection, antioxidant, mitochondrial network, mitochondrial resilience, neurodegenerative disease, 6-OHDA, MPP^+^

## Abstract

Parkinson’s disease (PD) is characterized by oxidative stress, mitochondrial dysfunction, and dopaminergic neuron loss, for which effective treatments remain unavailable. Here, we report CL0179, a fungal-derived decahydrofluorene alkaloid with antioxidant-associated neuroprotective properties, and evaluate its effects across cellular and animal PD models. CL0179 exhibited a favorable safety profile and protected SHSY5Y against 6-hydroxydopamine- (6-OHDA), rotenone-, and 1-Methyl-4-phenylpyridinium-iodide (MPP^+^)-induced neurotoxicity by preserving mitochondrial membrane potential and network integrity. Transcriptomic analyses revealed selective restoration of gene-expression programs associated with oxidative phosphorylation, mitochondrial bioenergetics, and stress adaptation disrupted by MPP^+^. CL0179 also enhanced SIRT1 activity under MPP^+^ stress, whereas pharmacological SIRT1 inhibition partially attenuated protection of mitochondrial membrane potential and cell viability. In *LRRK2*-G2019S astrocytes, CL0179 reduced ROS and α-synuclein accumulation and restored mitochondrial organization, while in human dopaminergic neurons, it attenuated toxin-induced mitochondrial depolarization and preserved neuronal architecture. To overcome the low production of CL0179, we generated the structurally related analogue CL0670. Both compounds crossed the blood–brain barrier and protected mouse primary cortical neurons, while CL0670 improved motor deficits in a 6-OHDA mouse model. Collectively, these compounds promote mitochondrial resilience and stress-adaptive neuroprotection, supporting their potential for PD and related neurodegenerative disorders.

## 1. Introduction

Parkinson’s disease (PD) is the second most prevalent neurodegenerative disorder, whose incidence increases significantly with age [1,2]. It is pathologically characterized by the progressive loss of dopaminergic neurons in the *substantia nigra pars compacta* and the accumulation of α-synuclein-containing inclusions known as Lewy bodies [3,4,5]. Although the exact etiology of the disease remains unclear, accumulating evidence indicates that mitochondrial dysfunction and oxidative stress are central contributors to PD pathogenesis [6,7,8]. Specifically, the impairment of mitochondrial complex I activity can lead to excessive production of reactive oxygen species (ROS), triggering oxidative damage, neuroinflammation, and ultimately neuronal death [9,10]. These pathogenic mechanisms highlight the urgent need for therapeutic strategies capable of protecting mitochondrial function and restoring cellular redox balance.

Natural compounds have shown significant pharmacological potential, including antioxidant, anti-inflammatory, and neuroprotective effects, positioning them as promising candidates for the development of novel therapeutics [11]. Preclinical studies have demonstrated that non-enzymatic antioxidants and dietary flavonoids can reduce ROS [12], stabilize mitochondrial function [13], chelate transition metals [14], modulate *NRF2* and *SIRT1* signaling [15] and attenuate α-synuclein accumulation and toxicity, mitigating the progressive loss of dopaminergic neurons [16]. However, translation to clinical efficacy has been limited by inadequate blood–brain barrier (BBB) penetration, poor bioavailability, heterogeneity of disease stage at intervention, and lack of validated oxidative biomarkers [17]. These limitations highlight the need to identify novel natural products and to unlock their clinical utility, offering a promising way towards true disease-modifying therapies.

To this end, beyond direct radical scavenging, increasing evidence suggests that effective neuroprotective strategies may depend on the engagement of endogenous stress-adaptive responses that preserve mitochondrial function and maintain cellular redox homeostasis. In this context, the concept of mitochondrial resilience, the capacity of mitochondria to adapt and recover from oxidative and bioenergetic stress [18,19], has recently emerged as a critical determinant of neuronal survival in neurodegenerative disorders. Therapeutic approaches capable of reinforcing stress-adaptive mechanisms while restoring mitochondrial fitness may therefore provide a more effective and durable strategy than conventional antioxidant supplementation alone.

In this work, we identified CL0179, a fungal-derived decahydrofluorene alkaloid, as a potent neuroprotective compound with therapeutic potential for PD. The compound displayed a favorable pharmacological safety profile, crossed the BBB and exerted robust protective effects across different toxin-induced and genetic PD models, including SH-SY5Y cells, mutant *LRRK2*-G2019S astrocytes, human dopaminergic neurons (hDAn), and primary mouse cortical neurons. Mechanistically, this compound preserved mitochondrial membrane potential (ΔΨm) and mitochondrial network integrity, reduced oxidative stress and α-synuclein accumulation, enhanced SIRT1 activity, and promoted endogenous stress-adaptive response programs rather than broad transcriptional reprogramming. To overcome the limited availability of CL0179, we developed the semisynthetic analogue CL0670 and evaluated its neuroprotective potential using in vitro and in vivo PD models, demonstrating functional protection against dopaminergic injury.

Collectively, these findings position natural decahydrofluorene alkaloids as promising neuroprotective scaffolds that promote mitochondrial resilience and engage stress-adaptive mechanisms, with a partial contribution of SIRT1, supporting their translational potential for PD and related neurodegenerative disorders.

## 2. Materials and Methods

### 2.1. Compounds

CL0179/CL0670: *Penicillium citrinum* HT-09B-VENT-WQ002 was isolated from soil and a sample was deposited at the Spanish Type Culture Collection (CECT) at the University of Valencia (CECT accession number 20769). Fermentation was conducted in 250 mL of media [2% oatmeal, 2% malt extract (Fisher Scientific, Madrid, Spain), 0.01% KH_2_PO_4_ (Scharlau, Barcelona, Spain), and 0.005% MgSO_4_ (Scharlau) (*w*/*v*) in tap water], under aerobic, mesophilic conditions for 7 days at 25 °C on a rotary shaker (200 rpm). The fermented broth was filtered through Radifil RW50 (Agrovin, León, Spain), and the resulting mycelial biomass was extracted with 2 L of 3:1 EtOAc (PanReac, Barcelona, Spain)/MeOH (PanReac) at 40–43 °C. The extract was fractionated by vacuum flash chromatography on silica gel using a stepwise gradient of hexane (PanReac)/EtOAc /MeOH at room temperature (RT), to finally yield 12 fractions (Fr. A to L). CL0179 was obtained from Fr. E by repeated purifications using HPLC (Prep 1100 system; Kinetex 5 µm EVO C18 100 Å, LC Column 100 × 21.2 mm; Agilent, Santa Clara, CA, USA), using a 25 min gradient from 40% to 100% aqueous MeOH at a flow rate of 16.61 mL/min. CL0670 was obtained by semisynthesis from CL0257, a compound structurally related to CL0179 obtained from Fr. B of the vacuum flash chromatography step. The precursor compound CL0257 was dissolved in CH_2_C_l2_ (PanReac) and 1.2 equivalents of metachloroperbenzoic acid (Sigma-Aldrich, St. Louis, MO, USA) at RT overnight. The reaction mixture was washed with a 5% NaHCO_3_ (PanReac) solution, neutralized with a saturated brine solution, and taken to dryness. The crude residue was crystallized from MeOH. Lyophilized CL0179 and CL0670 were stored at −20 °C. For in vitro experiments, the compounds were resuspended in sterile dimethyl sulfoxide sterile-filtered (DMSO; Sigma-Aldrich; 5 mM) before use. For the pharmacokinetics assay, in Cremophor:DMSO:H_2_O (10:10:80) and for the in vivo tests in Cremophor:EtOH:H_2_O (10:5:85).

Hydrogen peroxide solution (H_2_O_2_, Sigma-Aldrich): stored pure (9.79 M).

N-Acetyl-L-Cysteine (NAC; Sigma-Aldrich): stored as powder in the dark at 4 °C and resuspended in sterile water (H_2_O, Gibco; 100 mg/mL) by heating the mixture to 37 °C before use.

6-OHDA (Sigma-Aldrich): stored as powder, protected from light at RT. For in vitro experiments, resuspended in sterile H_2_O (50 mg/mL) before use, except for primary neuronal culture treatment in which it was resuspended in 0.15% ascorbic acid (100 mM) and for in vivo tests in 0.02% ascorbic acid (100 mM).

Rotenone (Sigma-Aldrich): resuspended in sterile DMSO (0.5 mg/mL) and stored at −20 °C in small aliquots.

MPP^+^ (Sigma-Aldrich): stored as powder, protected from light at RT and resuspended in sterile H_2_O (10 mg/mL) before use.

N-methyl-D-aspartate (NMDA; Sigma-Aldrich): resuspended in sterile H_2_O (10 mM) and stored at −20 °C in small aliquots.

Amyloid-beta fragment 25-35 (Sigma-Aldrich): resuspended in sterile H_2_O (1 mM), incubated at 37 °C for 72 h to allow for oligomerization, and stored at −20 °C in small aliquots.

### 2.2. In Vitro Safety Pharmacology Screening

Electrophysiological studies using the QPatch HT and IonFlux HT platforms, as well as the in vitro SafetyScreen™ panel, were performed by Eurofins Discovery, using 1 μM of the compounds.

### 2.3. Cell Culture

Human neuroblastoma cell line (SH-SY5Y): cells were cultured in Dulbecco’s Modified Eagle’s Medium (DMEM) [8.3 g DMEM powder (Sigma-Aldrich), 3.7 g sodium bicarbonate (Sigma-Aldrich), 1 g glucose (VWR), and 3.18 mL phenol red solution (Sigma-Aldrich) in a final volume of 1 L distilled water (pH 7.2)]. The medium was supplemented with 10% heat-inactivated fetal bovine serum (Fisher Scientific) and 4 mM L-glutamine (Sigma-Aldrich). Cells were sub-cultured every 3 days using Trypsin (Sigma-Aldrich).

Human induced pluripotent stem cells (hiPSC): line 1H was kindly provided by Dr. Lee (UTHealth Houston, TX, USA). Cells were maintained on a laminin-521 coating [Biolamina, Sundbyberg, Sweden; 0.5 μg/cm^2^ in DPBS (Dulbecco’s Phosphate-Buffered Saline; Gibco, Waltham, MA, USA)] using mTESR Plus medium (StemCell Technologies, Vancouver, BC, Canada) with 1% Penicillin/Streptomycin (P/S; Sigma-Aldrich). Cells were sub-cultured every 3 days using accutase (Sigma-Aldrich). Rock inhibitor (StemCell Technologies; 10 μM) was added to the medium on the day cells were subcultured.

Human astrocytes generated from patient-derived hiPSC (SP13) and control-derived hiPSC (SP09): cells were used as previously [20]. Cells at passage 4 were seeded onto Matrigel-coated (Corning, New York, NY, USA) culture plates and maintained in CNTF medium [Neurobasal (Gibco), 1× NEAA (Lonza, Basel, Switzerland), 2 mM Ultra Glutamine (Lonza), 1% P/S (Lonza), 1× B27 supplement (Gibco)] supplemented with 10 ng/mL CNTF (Peprotech, New Jersey, NJ, USA) for 14 days. Subsequently, the different experiments were performed. Half-medium changes were carried out every 5 days.

Differentiation of 1H hiPSC to generate hDAn: an optimized protocol, based on Nolbrant et al., 2017 [21], was carried out. Briefly, ventral-midbrain specification was achieved through dual SMAD inhibition using 10 μM SB431542 (Miltenyi Biotec, Bergisch Gladbach, Germany) and 100 ng/mL Noggin (Miltenyi Biotec) during the early differentiation, which promotes neural induction by blocking BMP and TGF-β pathways. This was followed by a finely controlled caudalization using 0.6 μM of the GSK-3 inhibitor and WNT pathway agonist CHIR99021 (Sigma-Aldrich) and ventralization through floor-plate induction using 300 ng/mL recombinant Sonic Hedgehog (SHH-C24II; Miltenyi Biotec). All four factors were added in N2 medium, consisting of 1:1 DMEM/F12 (Sigma-Aldrich) and Neurobasal media (Gibco), supplemented with 1× N2 (Gibco), 2 mM L-Glutamine and 0.2% P/S. Half-medium changes were performed every 2 days by removing 50% of the N2-supplemented medium and replacing it with an equal volume of fresh N2-supplemented medium. After 11 days, replating was carried out. To achieve the correct maturation of the ventral-midbrain progenitor cells, they were cultured in B27 medium consisted of Neurobasal media supplemented with 1× B27 w/o Vitamin A (Gibco), 2 mM L-Glutamine, 100 ng/mL FGF8b (Miltenyi Biotec), 20 ng/mL BDNF (Miltenyi Biotec), 0.2 mM Ascorbic acid (Sigma-Aldrich) and 0.2% P/S, from day 11 to day 16. Medium changes were performed every 2 days by removing B27-supplemented medium and replacing it with fresh B27-supplemented medium. On day 16, a second replating was performed. To differentiate the mature ventral-midbrain progenitors into mature dopaminergic neurons, cells were cultured in B27 medium supplemented with 1× B27 w/o Vitamin A, 2 mM L-Glutamine, 20 ng/mL BDNF, 0.2 mM Ascorbic acid, 10 ng/mL GDNF (R&D Systems), 500 μM cAMP (Sigma-Aldrich), 1 μM DAPT (R&D Systems) and 0.2% P/S until day 50. Rock inhibitor (10 μM) was added to the medium on the day cells were replated. Differentiation was monitored by immunofluorescence as described previously [22]. Mature ventral-midbrain progenitors (day 16) or hDAn (day 50) were fixed with 3.7% paraformaldehyde (PFA; Sigma-Aldrich) for 15 min at RT. Rabbit anti-LMX1B (Proteintech, Rosemont, IL, USA; 1:500) and goat anti-FOXA2 (R&D-Systems; 1:200) primary antibodies were employed to characterize mature ventral-midbrain progenitors, and rabbit anti-TH (Abcam, Cambridge, UK; 1:200) was used for hDAn. The secondary antibodies used were Alexa Fluor 568 anti-goat IgG (Invitrogen, Carlsbad, CA, USA; 1:1000) and Alexa Fluor 488 anti-rabbit IgG (Invitrogen; 1:1000). Confocal microscopy images were obtained in a Zeiss LSM800 Confocal Laser Scanning Microscope (Zeiss, Oberkochen, Germany) using 10× Plan-Apo/0.45 numerical aperture air objective and 25× Plan-Apo/0.8 numerical aperture oil objective.

Culture of Primary Cortical Neuron: neuronal cultures were prepared from C57BL/6J mouse embryo (E14.5) cortices. Neurons were seeded on pretreated poly-D-lysine (Sigma Aldrich; 10 μg/mL) in Neurobasal-A medium (Invitrogen) supplemented with 2% B27 (Invitrogen), 5.5 mM glucose (Sigma Aldrich), 0.25 mM pyruvate (Sigma Aldrich), 2 mM L-glutamine (Gibco), and antibiotics (penicillin G 100 U/mL; streptomycin 100 μg/mL; amphotericin B 0.25 μg/mL; Sigma-Aldrich). Medium was replaced every 3 days. Experiments were carried out on day 9–10.

All cell lines, except astrocytes, were cultured at 37 °C in a humidified atmosphere containing 5% CO_2_. Astrocytes were cultured at 37 °C in a humidified atmosphere containing 5% O_2_ and 5% CO_2_.

### 2.4. Cell Viability Assays

In all cases, except for primary cortical neurons, cell viability was quantified by the 3-(4,5-Dimethyl-2-thiazolyl)-2,5-diphenyl-2H-tetrazolium bromide (MTT)-based assay as described previously [23]. Absorbance was measured at 570 nm using a microplate Synergy HT reader (Agilent), using 630 nm as a reference wavelength for background correction. To assess the cytotoxic effect of compounds, cells were treated with CL0179 (0.05–5 μM), NAC (1 and 5 mM) or vehicle (sterile-filtered DMSO) for 72 h (96 h in the case of astrocytes) followed by the MTT assay. To determine their neuroprotective effect, cells were pre-treated with CL0179 (1 and 5 μM) or NAC (5 mM) for 24 h, followed by a 48 h co-treatment with the neurotoxins [(6-OHDA (75 μM); MPP^+^ (800 μM); Rotenone (5 μM) or H_2_O_2_ (200 μM)] and cell survival was evaluated by MTT assay.

Primary mouse cortical neuron viability was quantified using the fluorometric caspase-3 assay kit (Sigma Aldrich) as described previously [24]. To assess the cytotoxic effect of the compounds, cells were treated with CL0179 (0.01–10 μM), CL0670 (0.01–10 μM) or positive damage control (Amyloid-beta fragment 25-35; 10 μM) for 24 h. To determine their neuroprotective effect, primary neuronal cultures were treated in the presence or absence of NMDA (100 μM) or 6-OHDA (100 μM) for 15 min at 37 °C followed by a 4 or 24 h treatment, respectively, with CL0179 or CL0670 (0.1, 1 and 5 μM). Caspase-3 activity was determined as 7-amino-4-methylcoumarin (AMC) release rate extrapolating the slopes to those obtained from the AMC standard curve.

### 2.5. Mitochondrial Membrane Potential

ΔΨm was assessed using the JC-1 dye (Invitrogen). Cells were seeded in black, clear-bottom 96-well plates (Costar, New York, NY, USA). Cells were treated with CL0179 (1 and 5 μM) or NAC (5 mM) for 24 h prior to a 24 or 48 h neurotoxin challenge (6-OHDA (30 μM); MPP^+^ (100 μM); Rotenone (1 μM)). After treatment, cells were washed with Phosphate-Buffered Saline (PBS) and medium containing 2 μg/mL JC-1 was added. After a 30 min incubation at 37 °C, cells were washed twice with PBS and the fluorescence was measured with a microplate Synergy HT reader, using Ex/Em = 535/590 nm (J-aggregates) and Ex/Em = 485/530 nm (monomers). JC-1 is a cationic fluorescent probe that accumulates within mitochondria according to the mitochondrial membrane potential. In healthy mitochondria, with intact polarization, the dye forms red-emitting aggregates, whereas in depolarized mitochondria it remains in its monomeric form, showing green fluorescence. The lower ratio of red to green fluorescence indicates higher depolarization of the mitochondrial membrane potential.

### 2.6. Mitochondrial Morphology

MitoTracker Deep Red FM (Thermo Fisher Scientific, Waltham, MA, USA) was used for mitochondria labeling. Cells were treated with CL0179 (5 μM) or NAC (5 mM) for 24 h prior to a 12 h neurotoxin challenge (6-OHDA (75 μM); MPP^+^ (800 μM)), maintaining the compounds throughout the duration of the experiment. Cell culture medium with 250 nM of the fluorescent dye was added to the cells after treatment, and incubated for 30 min at 37 °C. Cells were washed twice with PBS and fixed with 3.7% PFA for 45 min at RT. Confocal microscopy images were obtained in a Zeiss LSM800 Confocal Laser Scanning Microscope using 63× Plan-Apo/1.40 numerical aperture oil objective at RT. Images were processed using the deconvolution method of ZEN BLUE 2.6 (Zeiss).

For mitochondria immunostaining, control (SP09), mutant (SP13), and CL0179-treated mutant (SP13 + CL0179; 1 μM; 96 h) astrocytes were fixed using 4% PFA for 20 min at RT and immunostaining was performed as previously described [25]. The following primary antibodies were used: guinea pig anti-GFAP (Synaptic systems; 1:1000) and rabbit anti-TOM20 (Santa Cruz, Starr County, TX, USA; 1:125), and the secondary antibodies: Alexa Fluor 647 anti-guinea pig IgG (Jackson ImmunoResearch, West Grove, PA, USA; 1:200) and Cy3 anti-rabbit IgG (Jackson ImmunoResearch; 1:200). Fluorescence microscopy images were obtained in a Zeiss Axio Imager M2 using the module Apotome 3 (Zeiss) with 40× Plan-Apo/1.40 and 63× Plan-Apo/1.40 numerical aperture oil objectives. Image quantification analysis was performed following the method published previously using the ImageJ software 1.54p [26].

### 2.7. SIRT1 Activity

SIRT1 enzymatic activity was measured using the SIRT1 Enzyme Activity Assay Kit (Abcam) according to the manufacturer’s instructions. SH-SY5Y cells were pre-incubated with CL0179 (5 μM) or vehicle control (DMSO) for 24 h, followed by a 24 h co-treatment with MPP^+^ (800 μM) prior to activity determination. Data were acquired every 2 min for 90 min using a microplate Synergy HT reader and the SIRT1 activity was expressed as the ratio of fluorescence intensity between sample versus control [27].

For SIRT1 inhibition, SH-SY5Y cells were subjected to the same incubation regimen in the presence of the specific SIRT1 inhibitor (EX-527, Fisher Scientific). Cells were pre-incubated with CL0179 (5 μM) or vehicle control (DMSO) in combination with Ex-527 (20 μM) for 24 h, followed by a 24 h co-treatment with MPP^+^ (800 μM) and Ex-527 (20 μM). Cell viability and ΔΨm were then evaluated according to the procedures described above. SIRT1 inhibition was confirmed by evaluating *TFAM* expression levels after treating SH-SY5Y cells with Ex-527 (20 μM) for 24 h. Total RNA from cultured cells was extracted using the RNeasy Mini Kit (Qiagen, Hilden, Germany). RNA yield and purity were assessed with a NanoDrop ND-100 spectrophotometer (Thermo Fisher Scientific). cDNA was subsequently synthesized using the High Capacity RNA-to-cDNA Kit (Applied Biosystems, Waltham, MA, USA). Quantitative real-time PCR (qRT-PCR) was performed using FastStart Universal SYBR Green Master Mix (Roche, Basel, Switzerland). Target-specific primer sequences are: TFM Fw: ATGGCGTTTCTCCGAAGCAT and TFAM Rv: TCCGCCCTATAAGCATCTTGA; 18S (housekeeping gene) Fw: GGCGCCCCCTCGATGCTCTTA and Rv: GCTCGGGCCTGCTTTGAACACTCT. The comparative threshold cycle method was used to quantify relative mRNA expression.

### 2.8. ROS Production

Intracellular ROS levels were quantified using the cell-permeant fluorescent probe 2′,7′-dichlorodihydrofluorescein diacetate (DCFH-DA; Sigma-Aldrich). For SH-SY5Y, cells were pre-treated with CL0179 (5 μM), CL0670 (5 μM) or NAC (5 mM) for 24 h, followed by a 12 h co-treatment with the damage (H_2_O_2_ (400 μM) or MPP^+^ (1500 μM)). For astrocytes, control (SP09) or mutant (SP13) astrocytes were treated with CL0179 (1 μM) or vehicle (DMSO) for 96 h. In both cases, after treatment, the medium was removed, cells were washed with PBS, and a medium containing 10 μM DCFH-DA was added. Cells were then incubated for 30 min at 37 °C and washed twice with PBS. The fluorescence intensity was detected using a fluorescence microplate Synergy HT reader setting Ex/Em = 485/530 nm.

### 2.9. Cell-Free Antioxidant Capacity

To measure the antioxidant scavenger activity of CL0179 and CL0670, the 2,2-diphenyl-1-picrylhydrazyl (DPPH; Sigma-Aldrich) radical scavenging assay was performed using TROLLOX (Sigma-Aldrich) as a positive control of scavenger activity. A 0.4 mM DPPH working solution was freshly prepared in anhydrous methanol and stored in the dark until use. In a 96-well microplate, 100 μL of different sample solutions TROLLOX (200–3.125 μM), CL0179, and CL0670 (200–0.625 μM) were mixed with 100 μL of DPPH working solution. The mixture was incubated at RT in the dark for 15 min. The absorbance of the reaction mixtures was measured at 517 nm using a microplate Synergy HT reader. The percentage of DPPH radicals was obtained as the ratio between absorbance of sample versus absorbance of DPPH without sample.

### 2.10. H_2_O_2_ Production

Primary neuronal cultures were exposed to NMDA (100 μM) for 15 min at 37 °C. Subsequently, cells were incubated for 4 h with CL0179 or CL0670 (0.1, 1, and 5 μM). H_2_O_2_ production was measured by the Amplex Red kit (Invitrogen), according to the manufacturer’s instructions [28].

### 2.11. α-Synuclein Accumulation

Control (SP09), mutant (SP13), or CL0179-treated mutant (SP13 + CL0179; 1 μM, 96 h) astrocytes were fixed using 4% PFA for 20 min at RT and immunostained for α-synuclein (BD Biosciences, Milpitas, CA, USA; 1:500) as described before [25], using Alexa Fluor 488 anti-mouse IgG (Jackson ImmunoResearch; 1:200) as secondary antibody. Confocal microscopy images were obtained in a Zeiss LSM800 Confocal Laser Scanning Microscope using 25× Plan-Apo/0.8 numerical aperture oil objective at RT. For quantification, images were first processed by applying a Gaussian blur filter (Sigma = 1), followed by threshold adjustment set to 25 for nuclear counting and 50 for α-synuclein quantification using the ImageJ software. Particle analysis was carried out with a minimum particle size of 0.1.

### 2.12. Immunostaining

SH-SY5Y cells were treated with CL0179 (5 μM) for 24 h prior to a 24 h MPP^+^-neurotoxin challenge (800 μM), maintaining the compounds throughout the duration of the experiment. Next, cells were washed twice with PBS, fixed with 3.7% PFA for 15 min at RT and immunostained for CASP-3 (BioNova, Fremont, CA, USA; 1:250) and TUJ-1 (Covance, New Jersey, NJ, USA; 1:500) as described before [22]. The secondary antibodies used were Alexa Fluor 546 anti-mouse IgG (Invitrogen; 1:1000) and Alexa Fluor 488 anti-rabbit IgG (Invitrogen; 1:1000). Confocal microscopy images were obtained in a Zeiss LSM800 Confocal Laser Scanning Microscope (Zeiss) using 25× Plan-Apo/0.8 numerical aperture oil objective.

### 2.13. Transcriptomic Analysis

SH-SY5Y cells were treated with CL0179 (5 μM) or vehicle (DMSO) for 24 h prior to a 24 h co-treatment with MPP^+^ (800 μM). Total RNA from three biological replicates per condition was isolated using QIAshredder (Qiagen) and the RNeasy mini kit (Qiagen). High-quality RNA samples were shipped to Novogene Co., Ltd. (Beijing, China) for library construction and high-throughput sequencing. mRNA library preparation was performed using poly(A) enrichment and sequencing was conducted on the Illumina NovaSeq X Plus Series platform (Illumina, San Diego, CA, USA) using a paired-end 150 bp configuration. A minimum sequencing depth yielding 12 Gb of raw data per sample was achieved. Mapping of the reads to the reference genome (*Homo sapiens* GRCh38), normalization to obtain the TPMs (transcript per million), and differential expression analysis were performed at the core service Bioinformática USAL (Universidad de Salamanca, Spain). The transcriptomic profiling is available at https://www.ncbi.nlm.nih.gov/geo/query/acc.cgi?acc=GSE345242 (accessed on 3 September 2026).

Sequence quality was assessed using FastQC software 0.12.0 [29] and transcript quantification was carried out with Salmon [30]. Differential expression analysis was performed with the edgeR v4 package [31], using a generalized linear model framework. Genes with an adjusted *p*-value (*p*-adj)  <  0.05 and a log2FoldChange (FC) < −0.5 or >0.5 were assigned as differentially expressed genes (DEGs). The web-tool ToppFun in ToppGene Suite [32] was used for functional enrichment analysis, applying the following options: “*p*-value Method = probability density function”, “multiple test correction = FDR”, “FDR cutoff < 0.05,” “Gene Limits 1 ≤ *n* ≤ 2000”. When summarizing lists of gene ontology (GO) terms, the web server REVIGO (http://revigo.irb.hr/. Accessed on 15 May 2026) [33] was used. The KEGG pathways network was generated via the Shiny Go web-tool (https://bioinformatics.sdstate.edu/go/. Accessed on 15 May 2026) [34].

### 2.14. In Vivo Studies

C57BL/6 mice (Jackson Laboratories) were purchased from Charles River Laboratories (Calco, Italy). After an acclimatization period of at least 5 days, the animals were handled to familiarize them to the operator.

All animal procedures complied with the current European and Spanish legislation (2007/526/EC and RD53/2013, respectively). The protocols were approved by the Bioethics Committees of the Universidad de Salamanca (Ref 1383).

#### 2.14.1. Pharmacokinetics

The pharmacokinetic study was performed by CERETOX (Parc Cientific de Barcelona) in CD1 female mice (8–9 weeks old) administering CL0179 and CL0670 (150 mg/kg) intraperitoneally. The concentrations of the compounds in plasma and brain were determined at different times per duplicate. Samples were analyzed using an Agilent Infinity II 1290 HPLC system coupled to an Agilent 6230 TOF mass spectrometer (Agilent). Brains from treated mice were homogenized in PBS and an aliquot (100 μL) was mixed with 15 μL acetonitrile, followed by 1 mL ethyl EtOAc. Plasma samples were extracted with EtOAc. After vortex and centrifugation, the organic phase was evaporated under a nitrogen stream, and the residue was reconstituted in MeOH. Chromatographic separation was performed using an Agilent Zorbax Eclipse Plus C18 Rapid Resolution HD column (2.1 × 50 mm, 1.8 μm, 100 Å; Agilent). The mobile phases consisted of water containing 0.1% formic acid and MeOH. The gradient program was as follows: 0–7 min, linear gradient from 20% to 100% MeOH; 7–9 min, 100% MeOH; followed by a 3 min post-run equilibration. The flow rate was 600 μL/min and the injection volume 2 μL. Mass spectrometric analysis was performed over an *m*/*z* range of 100–3200 using a fragmentor voltage of 100 V. Quantification was based on the major ion at *m*/*z* 221 [M–CH_3_O], with UV detection at 220 nm. The retention time of the compound was 3.37–3.38 min. The fundamental pharmacokinetic parameters were obtained from the non-compartmental analysis using PK Solutions 2.0.2.

#### 2.14.2. 6-OHDA Mouse Model

A toxin-induced mouse model of PD was generated by unilateral double intrastriatal injection of 6-OHDA. C57BL/6 male mice (12 weeks) were randomly assigned to three experimental groups. Control animals received two intrastriatal injections of saline 0.02% ascorbic acid and an intraperitoneal vehicle injection. The 6-OHDA group received two intrastriatal injections of 6-OHDA and an intraperitoneal vehicle injection. The 6-OHDA + CL0670 group received the same 6-OHDA lesion followed by intraperitoneal administration of CL0670 (75 mg/kg).

Stereotaxic injections were performed as previously [35]. Mice were placed in a stereotaxic alignment system (Model 1900; David Kopf Instruments, Tujunga, CA, USA) with digital read out (Wizard 550, Anilam, Coös County, NH, USA). Injections were performed into the striatum at coordinates: 0.3 and 1.0 mm anterior to bregma, 2.3 and 2.1 mm lateral to midline, and 2.9 mm ventral to dura, using a 5 µL Hamilton syringe (Microliter 65RN, Hamilton, NV, USA) with a 26 S needle (type 2 tip) [36]. The solutions (1.5 µL) were injected using a mini-pump (UltraMicroPump III, World Precision Instruments, Sarasota, FL, USA) and a digital controller (Micro4 UMC4; World Precision Instruments), at a rate of 0.3 μL/minutes during 5 min. The syringe was left in place for another 5 min before slowly retracting it to prevent reflux. Wounds were sutured, and animals were allowed to recover from anesthesia in cages placed on a 37 °C thermostatic plate (Plactronic Digital, 25 × 60, JP Selecta, Barcelona, Spain).

#### 2.14.3. Animal Behavior Tests

Animals were allowed to acclimate to the testing room for at least 45 min before each session. Behavioral performance was evaluated before surgery and 7 days after 6-OHDA injection by the rotarod test, the beam-balance test, the cylinder test, and the open field test. All behavioral tests were performed at the same time of day. Apparatuses were thoroughly cleaned between animals to avoid olfactory cues.

Rotarod test. Motor coordination and balance were assessed using the accelerating rotarod test (Model 47600, Ugo Basile, Varese, Italy). Animals were trained for 4 consecutive days before baseline measurement to establish stable performance. During the test, mice were placed on a rotating cylinder initially moving at 4 rpm. The rotation speed then increased at a constant acceleration of 8 rpm until reaching 40 rpm after 4.5 min. The latency to fall from the rod was recorded for each animal. Reduced latency was interpreted as impaired motor coordination and balance.

Beam-balance test. Motor coordination and balance were further assessed using the beam-balance test. Mice were trained for 2 consecutive days to traverse an elevated narrow beam (12 mm) to reach an enclosed escape platform containing bedding material. During the test, the latency to reach the escape platform was recorded as the main outcome measure.

Cylinder test. Forelimb-use asymmetry was assessed using the cylinder test. Mice were placed individually in a transparent cylinder with a diameter of 11 cm. Two mirrors were positioned behind the cylinder to allow simultaneous visualization from different angles. Animals were allowed to explore the novel environment freely, and the number of wall contacts performed with the ipsilateral and contralateral forelimbs was quantified during 10 rearing events. Forelimb asymmetry was expressed as the percentage of use of the affected hindlimb. A value close to 50% was considered representative of normal symmetric forelimb use.

Open field test. General locomotor activity, exploratory behavior, and bradykinesia-related parameters were evaluated using the open field test. Mice were placed individually in an ANY-box arena measuring 40 × 40 cm. Animals were allowed to explore the arena freely for 10 min. Total distance travelled and maximum speed were quantified using the ANY-maze tracking software (ANYmaze^®^, Stoeling Co., Wood Dale, IL, USA).

### 2.15. Statistical Analysis

Statistical analyses were performed using GraphPad Prism software 8.0.1. Values were expressed as mean ± standard error of the mean (SEM). Data distribution was analyzed in all cases, and statistical significance was determined using one-way ANOVA with Dunnett’s post hoc test. *p* < 0.05 was considered statistically significant.

## 3. Results

### 3.1. Discovery and Safety Pharmacology Profiling of a Novel Fungal-Derived Alkaloid

**Figure 1:**
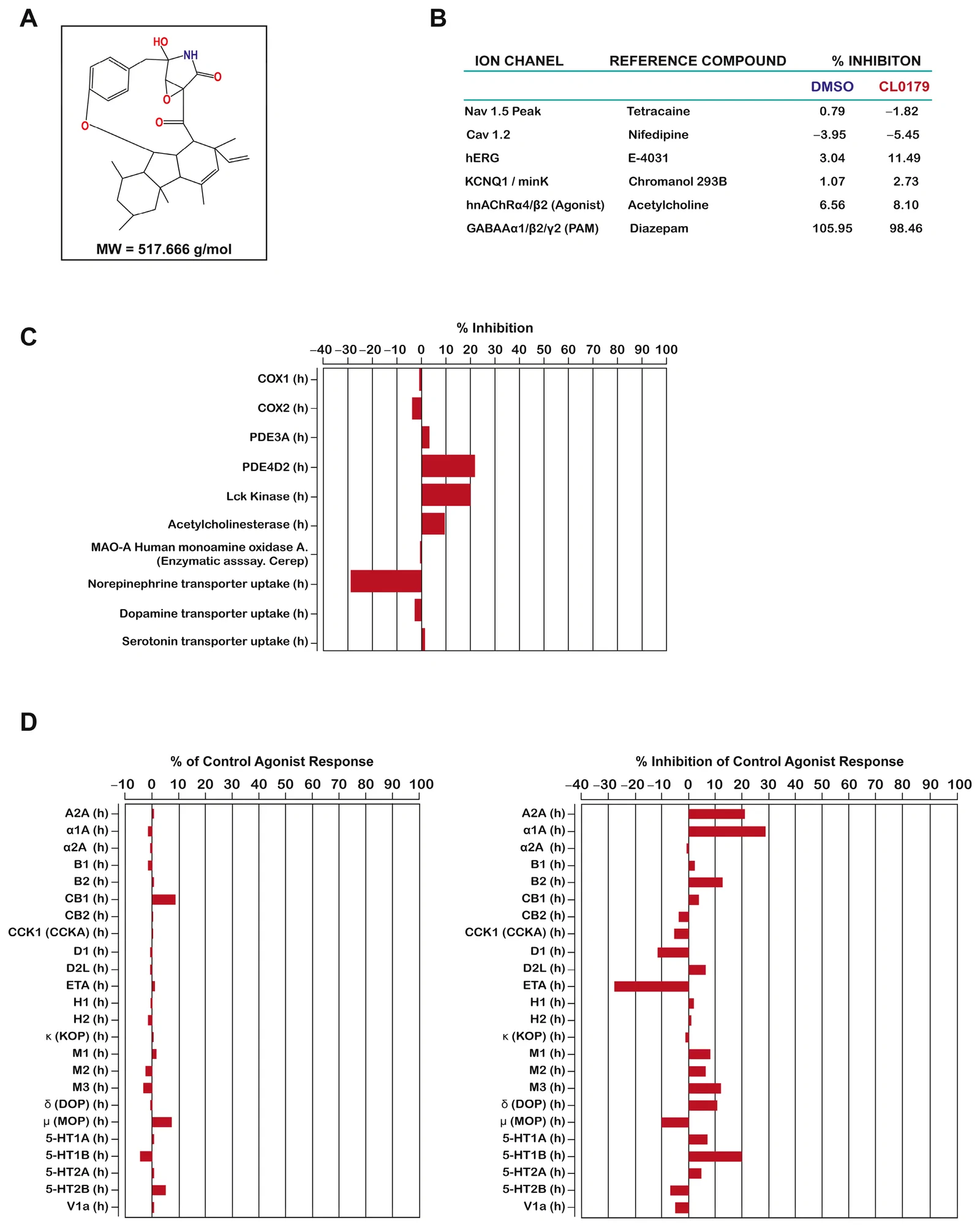
Structural elucidation and safety pharmacology profiling of the novel fungal-derived compound CL0179. (**A**) Chemical structure and molecular weight of CL0179. (**B**–**D**) Pharmacological safety studies. (**B**) Ion channel activity using QPatch HT and IonFlux HT electrophysiological platforms. (**C**) Enzyme, kinase, monoamine transporter, and GPCR functional assays, associated with neuroactive and inflammatory pathways. (**D**) Cellular receptor functional assays measuring agonist and antagonist activities across a broad range of GPCRs involved in neurotransmission, inflammation, cardiovascular regulation, and neuromodulatory signaling pathways.

An investigation of the culture broth of the fungal strain *Penicillium citrinum* HT-09B-VENT-WQ002 led to the isolation and identification of CL0179, a novel decahydrofluorene-class alkaloid responsible for the observed bioactivity. CL0179 presented a molecular weight of 517.666 g/mol and a molecular formula of C_32_H_39_O_5_N based on high resolution electrospray ionization mass spectrometry and analysis of ^1^H and ^13^C NMR data. The 2D structure of the compound was elucidated by analysis of ^1^H and ^13^C NMR spectra together with 2D NMR experiments, including COSY, gHSQC, and gHMBC (Figure 1A), as well as by comparing these data with those previously reported for related alkaloids [37,38].

To evaluate its pharmacological safety, electrophysiological studies (QPatch HT and IonFlux HT platforms) and a comprehensive in vitro analysis (SafetyScreen™ panel) were performed. CL0179 showed minimal effects across the ion channel panel, with inhibition values generally below biologically relevant thresholds, and it did not affect Nav1.5, Cav1.2, KCNQ1/minK, nicotinic acetylcholine receptor, or GABAAα1/β2/γ2 receptor activity (Figure 1B). Importantly, CL0179 showed only weak hERG inhibition (11.49%), indicating low predicted cardiotoxic liability (Figure 1B).

To assess pharmacological selectivity, the compound was screened against enzymes, kinases, monoamine transporters, and GPCR-related pathways. The results showed restricted pharmacological profiles, with moderate activity mainly toward the phosphodiesterase PDE4D2 and minimal effects on cyclooxygenase COX1/2, monoamine oxidase MAO-A, acetylcholinesterase, and major neurotransmitter-associated pathways (Figure 1C). Next, the agonist and antagonist activities across receptors involved in neurotransmission, inflammation, cardiovascular regulation, and neuromodulatory signaling were assessed (Figure 1D). The compound showed negligible agonist activity and only weak-to-moderate antagonist effects. The highest inhibition was observed at α1A-adrenergic and A2A-adenosine receptors, whereas CB1, opioid, muscarinic, and serotonergic receptors showed only minor responses, further supporting CL0179 limited off-target activity.

### 3.2. CL0179 Protects Against Oxidative Stress and Mitochondrial Neurotoxicity Through Mitochondrial Preservation

**Figure 2:**
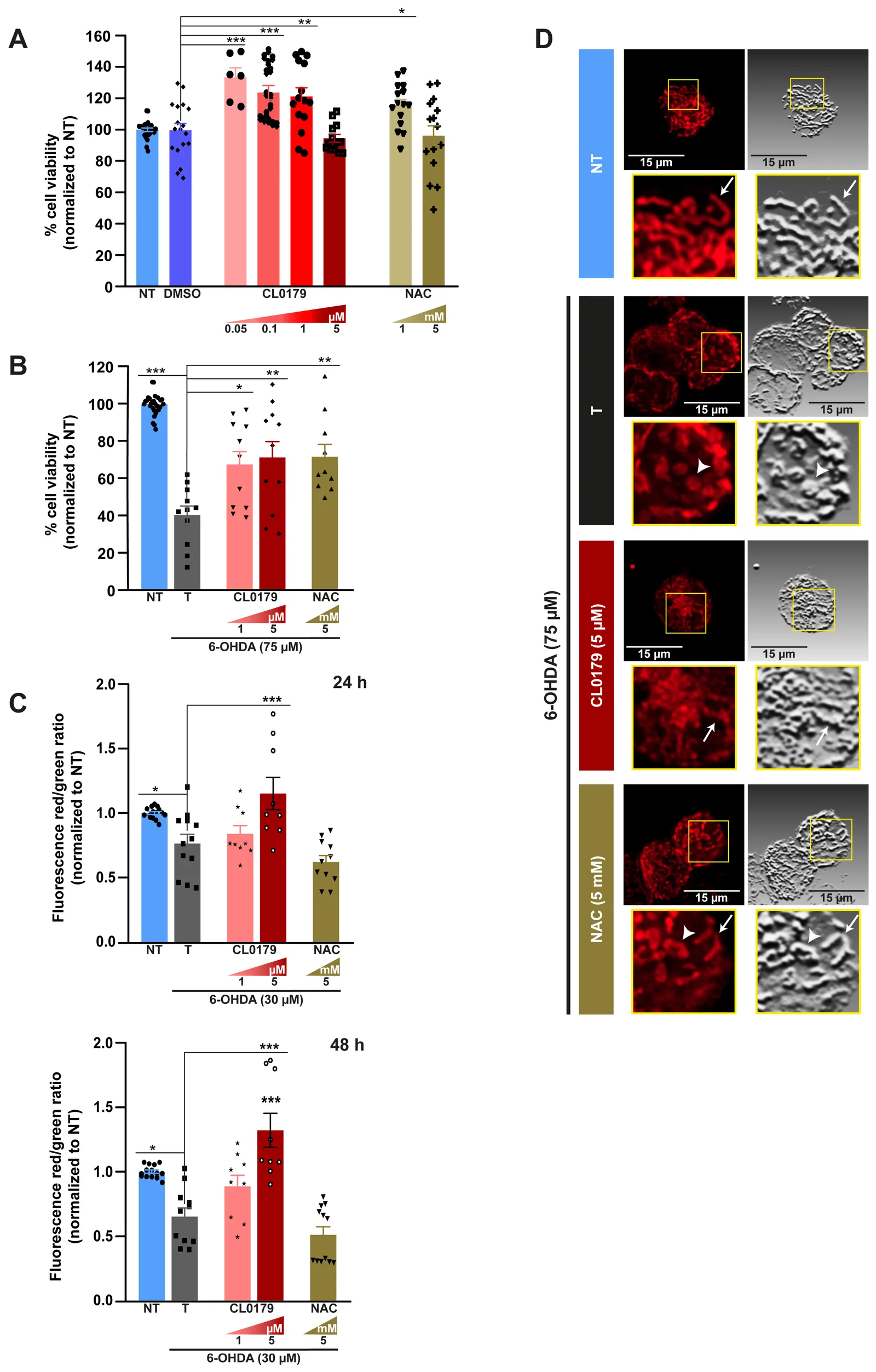
CL0179 attenuates 6-OHDA-induced neurotoxicity by preserving mitochondrial function and morphology in SH-SY5Y cells. (**A**) Cytotoxicity of CL0179 in SH-SY5Y cells (*n* = 3 independent experiments). (**B**) Neuroprotective effect of CL0179 against 6-OHDA-induced damage (*n* = 4 independent experiments). (**C**) CL0179 prevents 6-OHDA-induced depolarization of the mitochondrial membrane potential (*n* = 3 independent experiments). (**D**) Confocal microscopy images showing mitochondria morphology assessed by MitoTracker Deep Red FM staining. Scale bar: 15 μm. Yellow squares are magnified underneath. Grey-scale images were obtained by applying a bas-relief filter in Adobe Photoshop (Versión 24.0) solely for improved, qualitative-morphology assessment. Arrows: tubular mitochondria; Arrow heads: donut-like shaped mitochondria. NT: untreated; DMSO: dimethyl sulfoxide; NAC: N-Acetyl-L-Cysteine; T: 6-OHDA treatment. Data are expressed as mean ± SEM. * *p* < 0.05, ** *p* < 0.01 and *** *p* < 0.001 ((**A**) comparison to DMSO control cells; (**B**,**C**) comparison 6-OHDA-treated cells).

SH-SY5Y cells were used as a first-tier preclinical model for PD [39]. First, we assessed the intrinsic toxicity following exposure to increasing concentrations of CL0179. Like the neuroprotective control NAC, the compound lacked toxicity across the tested range (Figure 2A). Next, we evaluated the neuroprotective activity of CL0179 against complementary PD-relevant neurotoxic insults (6-OHDA, rotenone and MPP^+^). We began assessing its effect against 6-OHDA, a neurotoxin that induces extensive oxidative damage through ROS and quinone generation, affecting multiple cellular compartments in addition to mitochondria. As expected, 6-OHDA markedly reduced cell viability, while CL0179 partially restored cell survival, to levels comparable to NAC (Figure 2B).

To assess mitochondrial dysfunction, we quantified ΔΨm using JC-1. CL0179 significantly preserved the JC-1 fluorescence ratio at both 24 and 48 h, indicating attenuation of mitochondrial membrane depolarization, whereas NAC showed no significant effect (Figure 2C). MitoTracker imaging further supported these findings. Untreated cells displayed elongated and interconnected mitochondrial networks (Figure 2D, arrows), whereas 6-OHDA induced mitochondrial fragmentation and the appearance of ring-shaped (“donut-like”) mitochondria (Figure 2D, arrowheads), characteristic of mitochondrial stress and dysfunction [40,41]. In contrast, CL0179-cotreated cells retained elongated mitochondrial networks with reduced fragmentation (Figure 2D, arrows), outperforming the protective effect of NAC and indicating preserved mitochondrial integrity.

**Figure 3:**
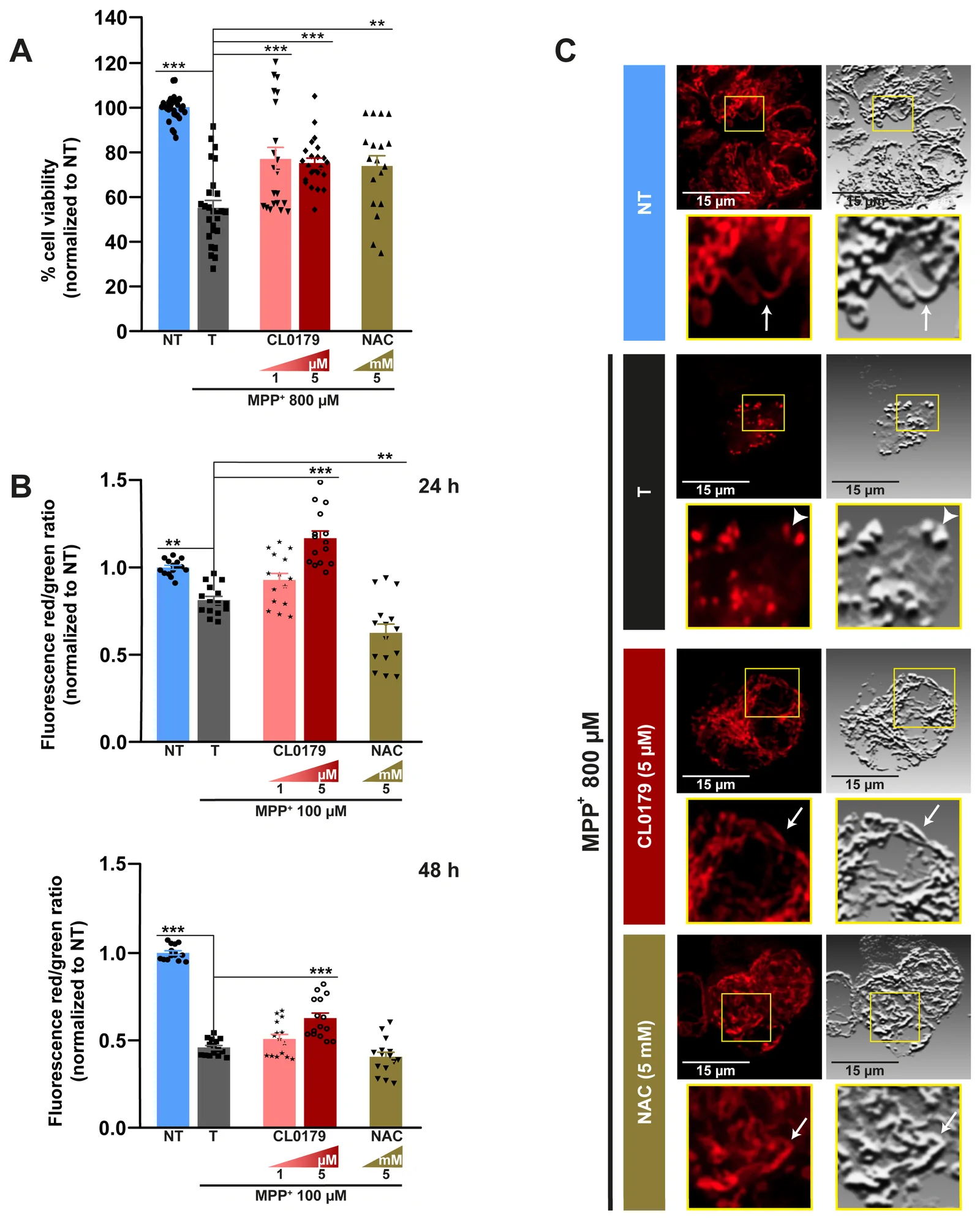
CL0179 preserves mitochondrial homeostasis. (**A**) Neuroprotective effect of CL0179 against MPP^+^-induced damage (*n* = 5 independent experiments). (**B**) CL0179 prevents MPP^+^-induced depolarization of the mitochondrial membrane potential (*n* = 5 independent experiments). (**C**) Confocal microscopy images of mitochondria morphology assessed by MitoTracker Deep Red FM staining. Scale bar: 15 μm. Yellow squares are magnified underneath. Grey-scale images were obtained by applying a bas-relief filter in Adobe Photoshop (Versión 24.0) solely for improved, qualitative-morphology assessment. Arrows: tubular mitochondria; Arrow heads: dot-like shaped mitochondria. NT: untreated; NAC: N-Acetyl-L-Cysteine; T: MPP^+^ treatment. Data are expressed as mean ± SEM. ** *p* < 0.01 and *** *p* < 0.001 ((**A**,**B**) comparison to MPP^+^-treated cells).

We then investigated whether this protection extended to rotenone and MPP^+^ models of primary mitochondrial dysfunction. Both neurotoxic insults inhibit mitochondrial Complex I through distinct mechanisms. Rotenone passively diffuses into cells and induces widespread mitochondrial dysfunction and oxidative stress, whereas MPP^+^ accumulates in mitochondria after cellular uptake, inducing severe bioenergetic failure, ATP depletion, mitochondrial membrane depolarization, and bioenergetic failure [42]. Together, these complementary models enabled us to determine whether CL0179 confers general antioxidant protection or directly preserves mitochondrial function under complex I-dependent stress. CL0179 provided modest but significant protection against rotenone-induced cytotoxicity, although its effect on mitochondrial damage was partial and transient, limited to the first 24 h, nevertheless exceeding NAC effect (Supplementary Figure S1A,B). However, CL0179 significantly protected against MPP^+^-induced cytotoxicity (Figure 3A) and preserved ΔΨm following MPP^+^ exposure at both time-points post-treatment (Figure 3B). Moreover, the compound prevented mitochondrial fragmentation (Figure 3C). MPP^+^-treated cells displayed abundant dot-shaped mitochondria (Figure 3C, arrowheads), whereas CL0179 and NAC-treated cultures retained elongated and interconnected mitochondrial networks (Figure 3C, arrows).

**Figure 4:**
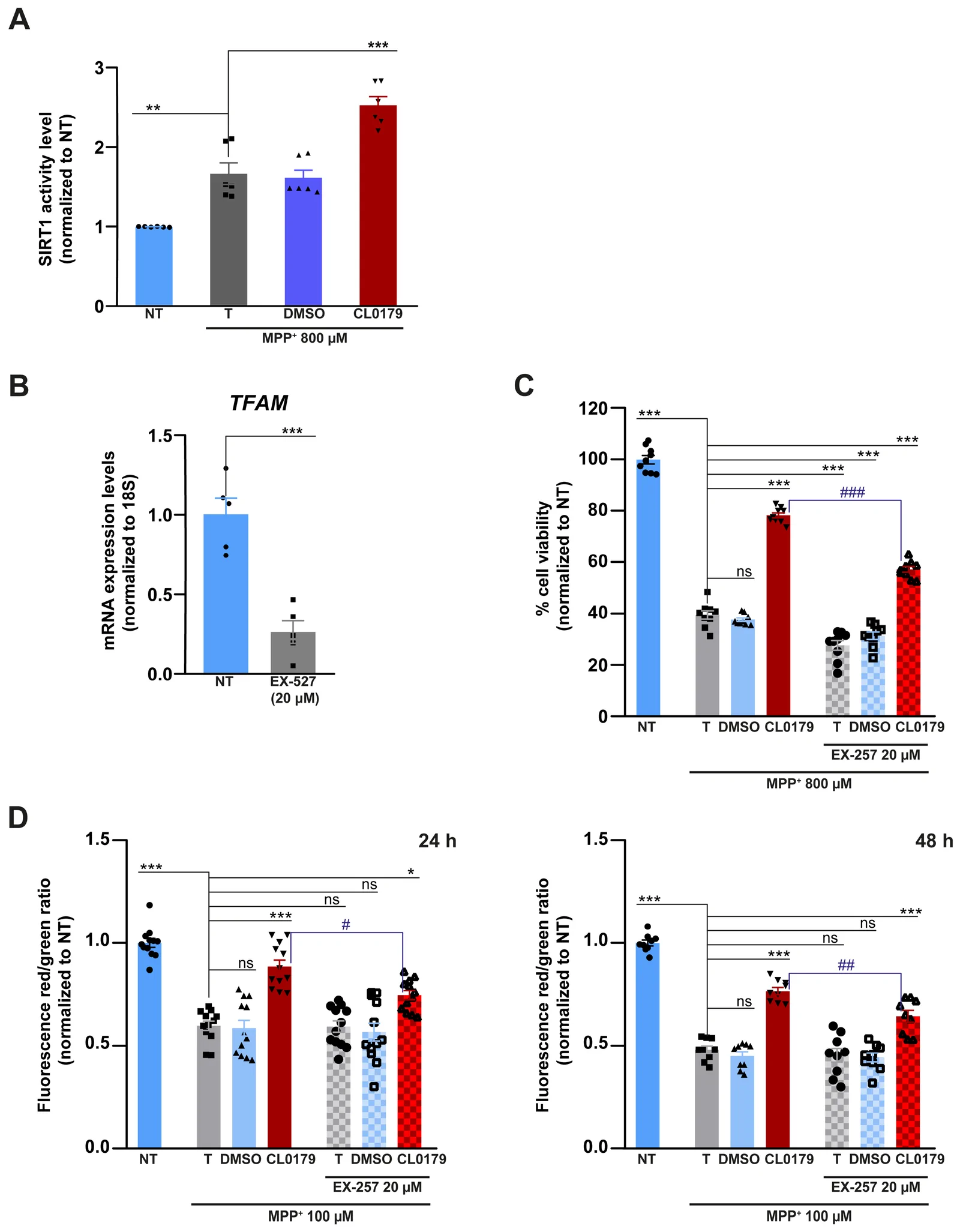
SIRT1 contributes to mitochondrial resilience in SH-SY5Y cells. (**A**) SIRT1 enzyme activity after MPP^+^ exposure. Data are expressed as the ratio of fluorescence intensity between samples versus control (*n* = 3 independent experiments). (**B**) Relative *TFAM* expression levels upon SIRT1 inhibition with EX-527 (*n* = 3 independent experiments). (**C**) Partial reversal of CL0179-mediated neuroprotection on cell viability by the SIRT1 inhibitor EX-527 (*n* = 3 independent experiments). (**D**) Attenuation of CL0179-driven preservation of mitochondrial membrane potential following SIRT1 blockade (*n* = 3 independent experiments). NT: untreated; DMSO: dimethyl sulfoxide; T: MPP^+^ treatment; ns: not significant. CL0179 5 μM. Data are expressed as mean ± SEM. *^,#^ *p* < 0.05, **^,##^ *p* < 0.01 and ***^,###^ *p* < 0.001((**A**) comparison to MPP^+^-treated cells; (**B**) comparison to non-treated cells; (**C**,**D**) * comparison to MPP^+^-treated cells, # comparison to CL0179-treated cells).

Given that the maintenance of interconnected mitochondrial networks relies on finely tuned stress-response programs, we hypothesized that the Cl0179 protective effect might engage SIRT1, a NAD^+^-deacetylase that is a key regulator of cellular adaptation to metabolic and oxidative stress through pathways controlling mitochondrial homeostasis, antioxidant responses and mitochondrial biogenesis [43]. Notably, CL0179 increased SIRT1 activity in MPP^+^-stressed cells (Figure 4A), suggesting the involvement of SIRT1-associated adaptive responses in its neuroprotective activity. To investigate this possibility, SIRT1 was pharmacologically inhibited with EX-527. *TFAM* expression was used as a downstream readout of the SIRT1–PGC-1α mitochondrial biogenesis axis. EX-527 significantly reduced *TFAM* mRNA levels, consistent with inhibition of the SIRT1–PGC-1α axis (Figure 4B). Importantly, EX-527 partially attenuated CL0179-mediated protection against MPP^+^-induced cytotoxicity (Figure 4C) and mitochondrial dysfunction, reducing cell viability and preserving ΔΨm (Figure 4D). These findings support a contribution of SIRT1 to the cytoprotective and mitochondria-preserving effects of CL0179, suggesting that SIRT1-associated adaptive mechanisms participate in mitochondrial resilience to MPP^+^-induced stress. However, it is not sufficient to explain the observed protection, suggesting that additional SIRT1-independent mechanisms may also contribute to its neuroprotective activity.

### 3.3. CL0179 Induces a Restricted Mitochondrial and Stress-Associated Transcriptional Response Following MPP^+^ Exposure

**Figure 5:**
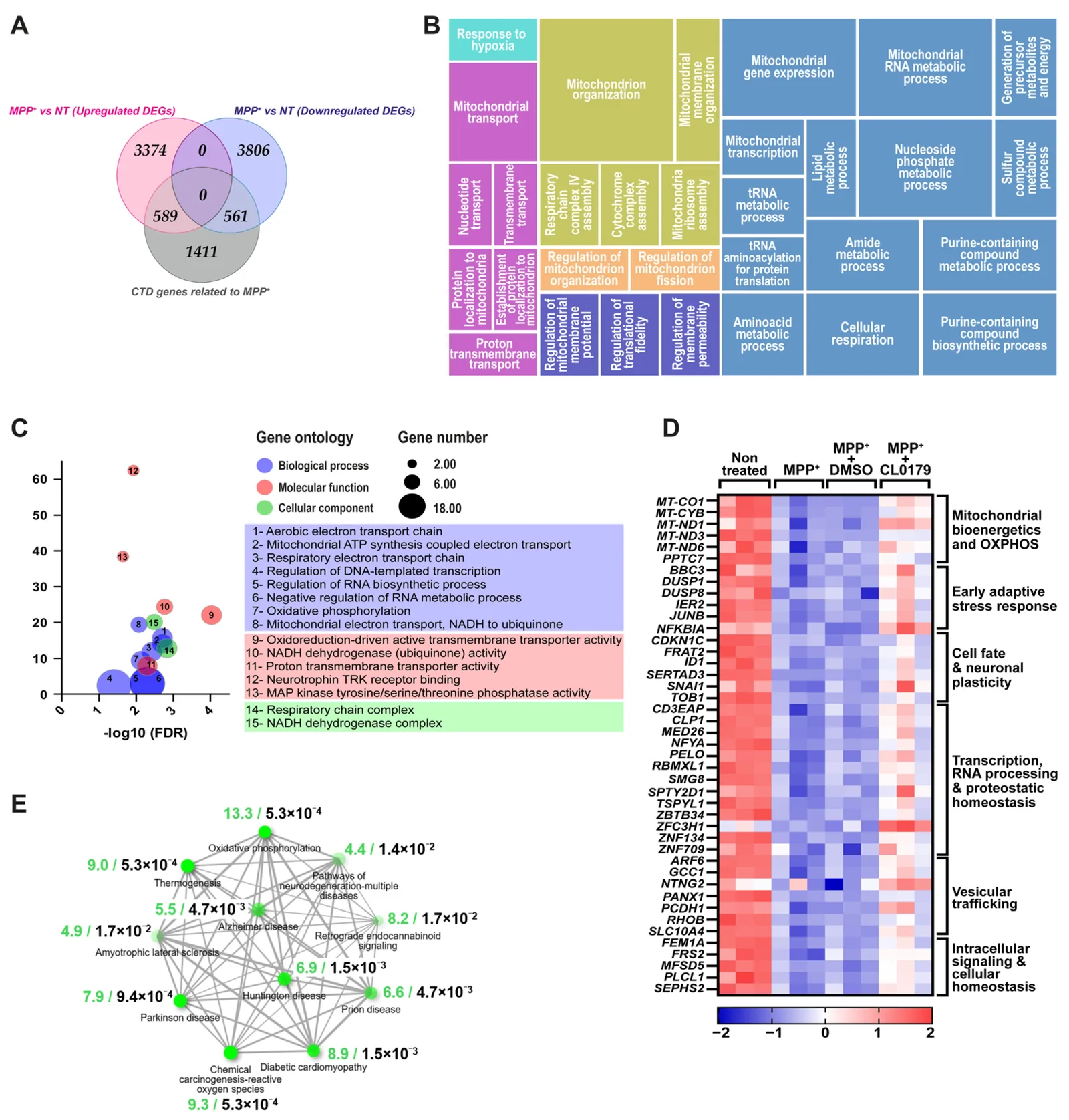
The candidate compound counteracts MPP^+^-induced transcriptional deregulation through mitochondria-centered mechanisms in SH-SY5Y cells. (**A**) Global analysis of the SH-SY5Y transcriptomic profile after MPP^+^-exposure. The Venn diagram illustrates the overlap between genes deregulated in our model and curated annotations for MPP^+^ at the Comparative Toxicogenomics Database (CTD). (**B**) Visualization of biological processes downregulated by MPP^+^ treatment, specifically associated with mitochondrial functions. The REVIGO tree map resulted from summarizing the top-100 GO IDs from the functional enrichment analysis. The size of the rectangles was adjusted according to the *p*-adj provided by ToppGene. (**C**) Functional enrichment analysis of DEGs upregulated by CL0179 treatment under MPP^+^ oxidative stress conditions. Node colors indicate the three gene ontology (GO) domains (Biological Process (blue), Molecular Function (pink), and Cellular Component (green)). Node size corresponds to the number of genes per term; the x-axis denotes fold enrichment and the y-axis indicates statistical significance expressed as log10(False Discovery Rate, FDR). (**D**) Heatmap showing the selective transcriptomic rescue driven by CL0179. The profile includes the downregulated DEGs in MPP^+^-treated cells whose expression was significantly upregulated upon co-treatment with CL0179. Z-score values from *n* = 3 independent biological replicates per condition. (**E**) KEGG pathways network analysis performed with the CL0179-upregulated DEGs.

To gain insight into the molecular mechanisms underlying CL0179 neuroprotective activity, we performed bulk RNA-seq on SH-SY5Y exposed to MPP^+^ and treated with the compound or DMSO. We first validated whether the MPP^+^-transcriptional response recapitulated established MPP^+^-neurotoxicity signatures. Differential expression analysis identified a wide transcriptomic deregulation following MPP^+^ treatment (3963 upregulated and 4367 downregulated DEGs). When comparing those DEGs against the MPP^+^-associated genes in the Comparative Toxicogenomics Database (CTD) [44], we identified 1150 significantly altered genes out of 2561 MPP^+^-associated genes (Figure 5A), demonstrating strong concordance with the established MPP^+^ transcriptional signature.

To further dissect the specific mitochondrial impact of the neurotoxin, the downregulated DEG list was cross-examined with the Broad Institute’s MitoCarta database [45]. Notably, 18.7% of all annotated mitochondrial genes in MitoCarta were significantly downregulated in our dataset. Accordingly, functional annotation of our gene subset highlighted prominent terms related to mitochondrion organization, respiratory chain complex assembly, mitochondrial RNA metabolic process, or regulation of ΔΨm, among others, indicating profound impairment of mitochondrial function (Figure 5B). In parallel, stress-responsive and apoptotic genes were highly significantly up-regulated, including *HSPA1B*, *DDIT3/CHOP*, *ATF4*, and *BAX* (Supplementary Materials), altogether consistent with activation of unfolded protein response and apoptotic pathways. Genes associated with oxidative stress adaptation such as *NQO1* were also altered, further supporting an extensive mitochondrial and redox imbalance in MPP^+^-treated cells. Altogether, these results confirmed that our experimental model faithfully recapitulated the molecular hallmarks of PD pathogenesis [46,47,48,49,50].

To identify molecular pathways linked to the neuroprotective effect of CL0179, we compared the MPP^+^-transcriptomic profiles with those obtained from cells co-treated with CL0179. Co-treatment with CL0179 modulated a targeted set of genes, with differential expression analysis identifying 90 DEGs, of which 83 were upregulated and 7 downregulated. This suggested that the molecule exerts a refined regulatory effect rather than a broad and less-specific transcriptomic rewiring. To rule out vehicle-induced artifacts, cells treated with MPP^+^ and DMSO were compared against the MPP^+^ alone group, yielding only one DEG, which was excluded from further analysis.

Given that the major therapeutic aim of a novel compound should be to counteract the neurotoxic effects on cellular functions, we focused on DEGs that displayed a directional reversal toward basal expression levels, thus profiling a rescue signal. Functional enrichment analysis of the upregulated DEG list uncovered several “mitochondria-centered” biological processes, molecular functions, and cellular components (Figure 5C), indicating that CL0179 elicits a counter-regulatory response, with the upregulation of core bioenergetic processes. Despite the relatively small gene count per term (typically 4–6 genes), the terms exhibited high specificity, with a median fold enrichment of 14. Among the top significantly enriched terms were aerobic electron transport chain, mitochondrial ATP synthesis coupled electron transport, NADH dehydrogenase activity, or the respiratory chain complex. Concurrently, the compound modulated crucial neuroprotective and survival pathways, as evidenced by the significant enrichment of neurotrophin TRK receptor binding and MAP kinase tyrosine/serine/threonine phosphatase activity.

To pinpoint the specific transcriptomic targets effectively rescued by CL0179, we analyzed the intersection of genes significantly downregulated by MPP^+^ and upregulated by CL0179 cotreatment (Figure 5D). The core of this rescue signature was driven by the respiratory chain and mitochondrial genome transcripts *MT-CO1*, *MT-CYB*, *MT-ND1*, *MT-ND3*, and *MT-ND6*, all of which are closely linked to mitochondrial bioenergetic function. In parallel, several stress-adaptive and survival-associated genes also showed expression changes toward basal levels. Together, these findings suggest that, rather than broadly reversing MPP^+^-induced transcriptional disruption, CL0179 selectively modulates a restricted set of mitochondrial and stress-responsive genes associated with cellular adaptation to bioenergetic stress.

Finally, we contextualized the CL0179-driven transcriptomic changes within broader functional pathways such as KEGG, using the complete upregulated DEG list (Figure 5E). In agreement with the GO enrichment findings, the network analysis prominently featured the “Oxidative phosphorylation” node in connection with PD. Notably, this analysis revealed a significant clustering around “Pathways of neurodegeneration-multiple diseases”, with interconnecting nodes not only for PD but also for Huntington or Alzheimer’s disease between others, collectively suggesting that CL0179 selectively counteracts MPP^+^-induced transcriptional alterations associated with mitochondrial bioenergetics and stress adaptation, pathways broadly implicated across neurodegenerative disorders.

### 3.4. CL0179 Prevents α-Synuclein Accumulation and Preserves Mitochondrial Network Integrity in LRRK2-G2019S PD-Derived Astrocytes and Attenuates Toxin-Induced Mitochondrial Depolarization in Human Dopaminergic Neurons

**Figure 6:**
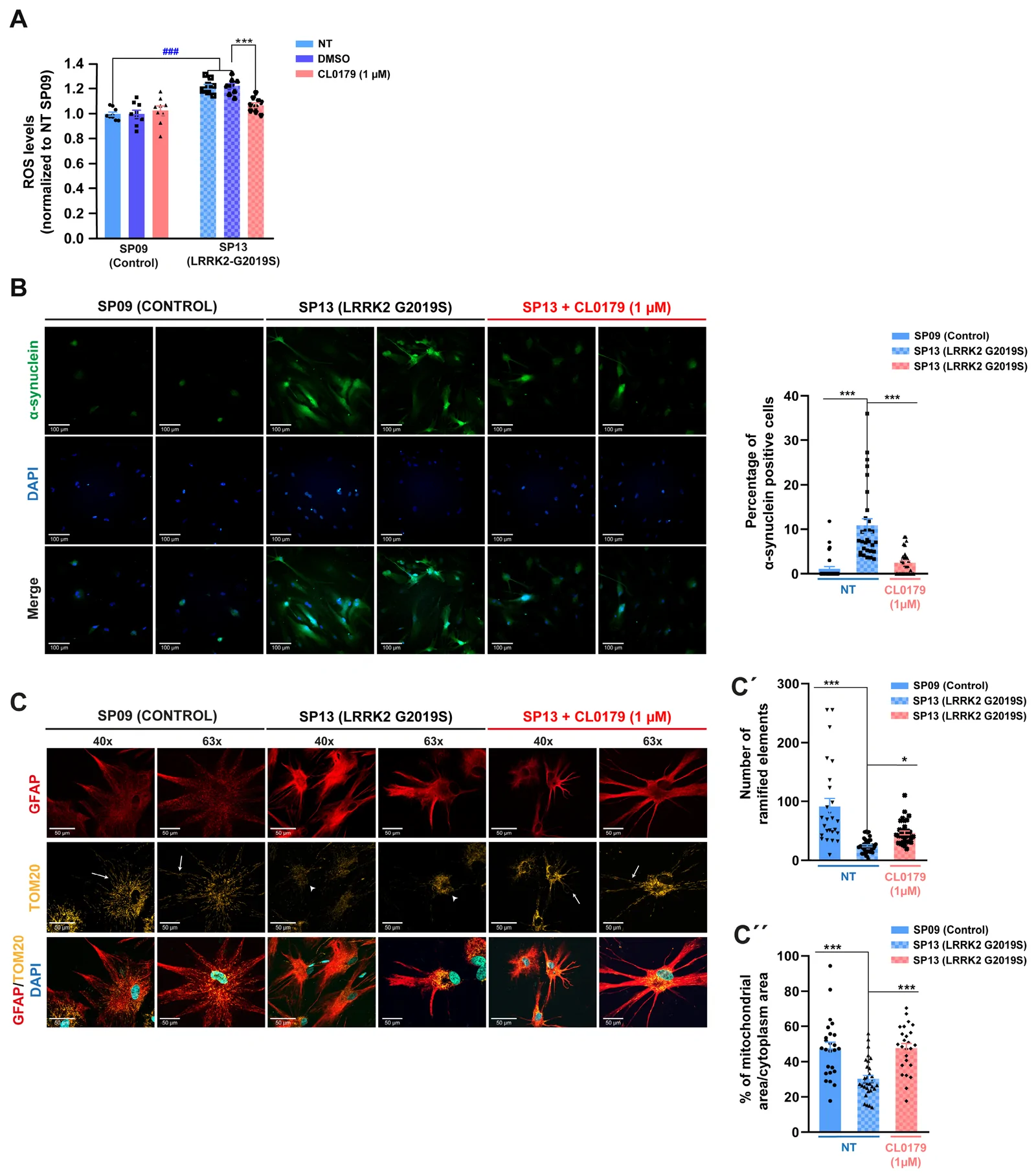
CL0179 prevents α-synuclein accumulation and preserves mitochondrial network integrity in LRRK2-G2019S PD-derived astrocytes. (**A**) CL0179 effect on reactive oxygen species (ROS) levels in control (SP09) or mutant (SP13) astrocytes. (*n* = 3 independent experiments). (**B**) Immunostaining of α-synuclein in control (SP09), mutant (SP13) or CL0179-treated mutant (SP13 + CL0179) astrocytes (α-synuclein in green, and nuclei-DAPI in blue). Scale bar: 100 μm. Percentage of α-synuclein-positive cells (*n* = 3 independent experiments). (**C**) Immunostaining of GFAP and TOM20 in astrocytes under three conditions: control (SP09), mutant (SP13), and CL0179-treated mutant (SP13 + CL0179) (GFAP in red, TOM20 in yellow and nuclei-DAPI in blue). Scale bar: 50 μm. Arrows: cytoplasmic mitochondria networks; Arrow heads: fragmented perinuclear mitochondria. (**C′**) Quantitative morphometric analysis of mitochondrial network connectivity indicated by the number of ramified elements (*n* = 25 cells). (**C″**) Quantitative morphometric analysis of mitochondrial footprint (*n* = 25 cells). ET: wild type; NT untreated cells; DMSO: dimethyl sulfoxide. Data are expressed as mean ± SEM. * *p* < 0.05, *** *p* < 0.001 and ^###^ *p* < 0.001 ((**A**) * comparison to mutant (SP13 + DMSO) astrocytes, # comparison to non-treated control (SP09) astrocytes; (**B**–**C″**) comparison to non-treated mutant (SP13) astrocytes.

To further substantiate our results in a translationally relevant setting, we evaluated the effect of CL0179 in a human genetic PD model using *LRRK2*-G2019S mutant astrocytes (SP13) and healthy controls (SP09) [20,25]. Previous studies have shown that *LRRK2*-PD astrocytes fail to provide full neurotrophic support to hDAn and display atrophic morphology and altered mitochondrial function as well as metabolic alterations [51,52]. The *LRRK2* G2019S mutation is the most common cause of monogenic PD. This autosomal dominant gain-of-function variant, characterized by high but incomplete penetrance, is located in the kinase domain and causes sustained kinase hyperactivation, leading to defects in cellular trafficking, lysosomal and mitochondrial dysfunction, and neuroinflammation [53,54]. In consonance, LRRK2-PD astrocytes fail to provide full neurotrophic support to hDAn and display atrophic morphology and altered mitochondrial function as well as metabolic alterations [49,52]. As expected, *LRRK2*-G2019S astrocytes displayed significantly elevated ROS levels, reflecting their enhanced oxidative stress status (Figure 6A). At non-toxic concentrations (Supplementary Figure S2A), CL0179 significantly reduced ROS accumulation in SP13 astrocytes to levels comparable to those observed in control cells (Figure 6A).

Strikingly, the marked α-synuclein accumulation observed in *LRRK2*-PD astrocytes was dramatically reversed following CL0179 treatment (Figure 6B), demonstrating the compound’s robust ability to reduce disease-associated α-synuclein accumulation. Moreover, TOM20 immunostaining combined with mitochondrial morphometric analysis revealed that the mitochondrial abnormalities present in *LRRK2*-PD astrocytes were substantially ameliorated by CL0179 treatment. Control astrocytes exhibited elongated and interconnected mitochondrial networks distributed throughout the cytoplasm (Figure 6C, arrows), whereas *LRRK2*-PD astrocytes displayed fragmented mitochondria clustered in the perinuclear region (arrowheads) together with reduced network connectivity (Figure 6C′). In addition, *LRRK2*-PD astrocytes exhibited a significant reduction in mitochondrial footprint, defined as the total cellular area occupied by mitochondria (Figure 6C″). These alterations, which are associated with oxidative stress and mitochondrial dysfunction, were significantly rescued by CL0179 treatment (Figure 6C′,C″), indicating that the compound can counteract disease-associated abnormalities in a genetically driven cellular context.

**Figure 7:**
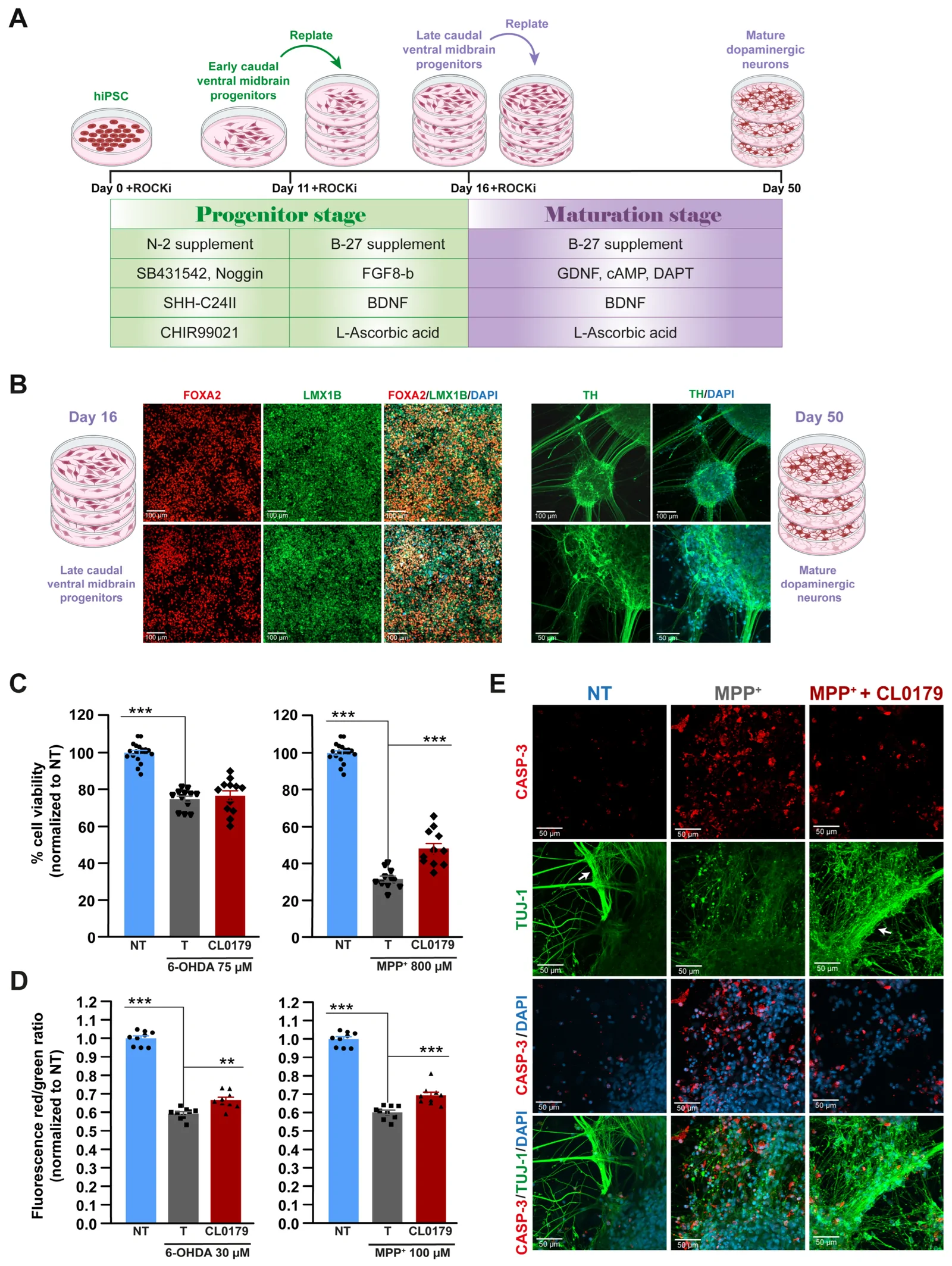
CL0179 attenuates MPP^+^-induced mitochondrial depolarization and preserves neuronal architecture in human dopaminergic neurons derived from human induced pluripotent cells (hiPSCs). (**A**) 1H hiPSCs optimized differentiation protocol based on the one previously described by [21]. (**B**) Immunostaining of mature ventral-midbrain progenitors (FOXA2 in red, LMX1b in green and nuclei-DAPI in blue) and mature dopaminergic neurons (TH in green and nuclei-DAPI in blue). Scale bar 100 μm (**C**) Neuroprotective effect of CL0179 against 6-OHDA- and MPP^+^-induced damage (*n* = 4 independent experiments). (**D**) Depolarization of the mitochondrial membrane potential under 6-OHDA- and MPP^+^-induced damage (*n* = 3 independent experiments). (**E**) CL0179 preserves neuronal architecture and limits MPP^+^-induced neuronal damage in human dopaminergic neurons (Cleaved Caspase-3 in red, TUJ-1 in green and nuclei-DAPI in blue). Arrows correspond to TUJ1-positive neuronal networks with prominent axonal bundles. Scale bar: 100 μm. NT untreated cells; T: 6-OHDA or MPP^+^ treatment. CL0179 5 μM. Data are expressed as mean ± SEM. ** *p* < 0.01 and *** *p* < 0.001 ((**C**,**D**) comparison to 6-OHDA or MPP^+^-treated cells).

Building upon the translational relevance of these findings, we extended our investigation to hDAn, the primary neuronal population affected in PD [55,56]. We generated hDAn from hiPSCs adapting a protocol from [21] (Figure 7A). After 16 days of differentiation, cultures were enriched in FOXA2^+^/LMX1B^+^ ventral midbrain progenitors, while by day 50, most cells corresponded to mature tyrosine hydroxylase-positive (TH^+^) hDAn (Figure 7B). TH is the rate-limiting enzyme in dopamine biosynthesis. Disruption of its stability, activity, or axonal transport causes dopamine deficiency, leading to impaired neuronal function, reduced plasticity, and motor deficits characteristic of PD [57,58]. CL0179 showed no intrinsic toxicity in either hiPSCs, ventral midbrain progenitors nor hDAn (Supplementary Figure S2B–D). Consistent with the increased vulnerability of hDAn to oxidative and mitochondrial stress [59], CL0179 neuroprotection was less pronounced than in SH-SY5Y cells and mainly noticeable after treatment with MPP^+^ (Figure 7C). Importantly, co-treatment with CL0179 mitigated the decrease in the JC-1 ratio induced by 6-OHDA and MPP^+^ (Figure 7D).

To visualize CL0179 neuroprotection, hDAn cultures were immunostained for TUJ1 and cleaved caspase-3 after the indicated treatments (Figure 7E). Representative confocal images showed that non-treated cultures formed dense TUJ1-positive neuronal networks with prominent axonal bundles (arrow) interconnecting adjacent neuronal spheroids. In contrast, MPP^+^ treatment markedly increased cleaved caspase-3 immunoreactivity and disrupted the compact organization of these axonal bundles, which appeared looser, less organized, and less densely packed than in untreated cultures. CL0179 treatment attenuated caspase-3 activation and preserved the compact axonal bundle (arrow) organization and overall neuronal architecture. Altogether, these data support the neuroprotective value of CL0179 in a physiologically relevant hDAn model.

We next sought to investigate whether CL0179 could induce a resilience-related transcriptional state in the absence of neurotoxic stress. To this end, we analyzed hDAn exposed to CL0179 under basal conditions. Transcriptomic analysis revealed that CL0179 induced only minimal transcriptional alterations under physiological conditions, thereby maintaining a favorable safety profile. Comparisons between untreated, DMSO, and CL0179-treated hDAn yielded a negligible number of DEGs, suggesting that CL0179 neuroprotective effect is consistent with a context-dependent activity that becomes more apparent under cellular stress conditions.

### 3.5. A Scalable CL0179 Analogue, CL0670, Maintains Neuroprotective Activity in SH-SY5Y and Murine Primary Cortical Neurons and Improves Motor Function in a PD Mouse Model

**Figure 8:**
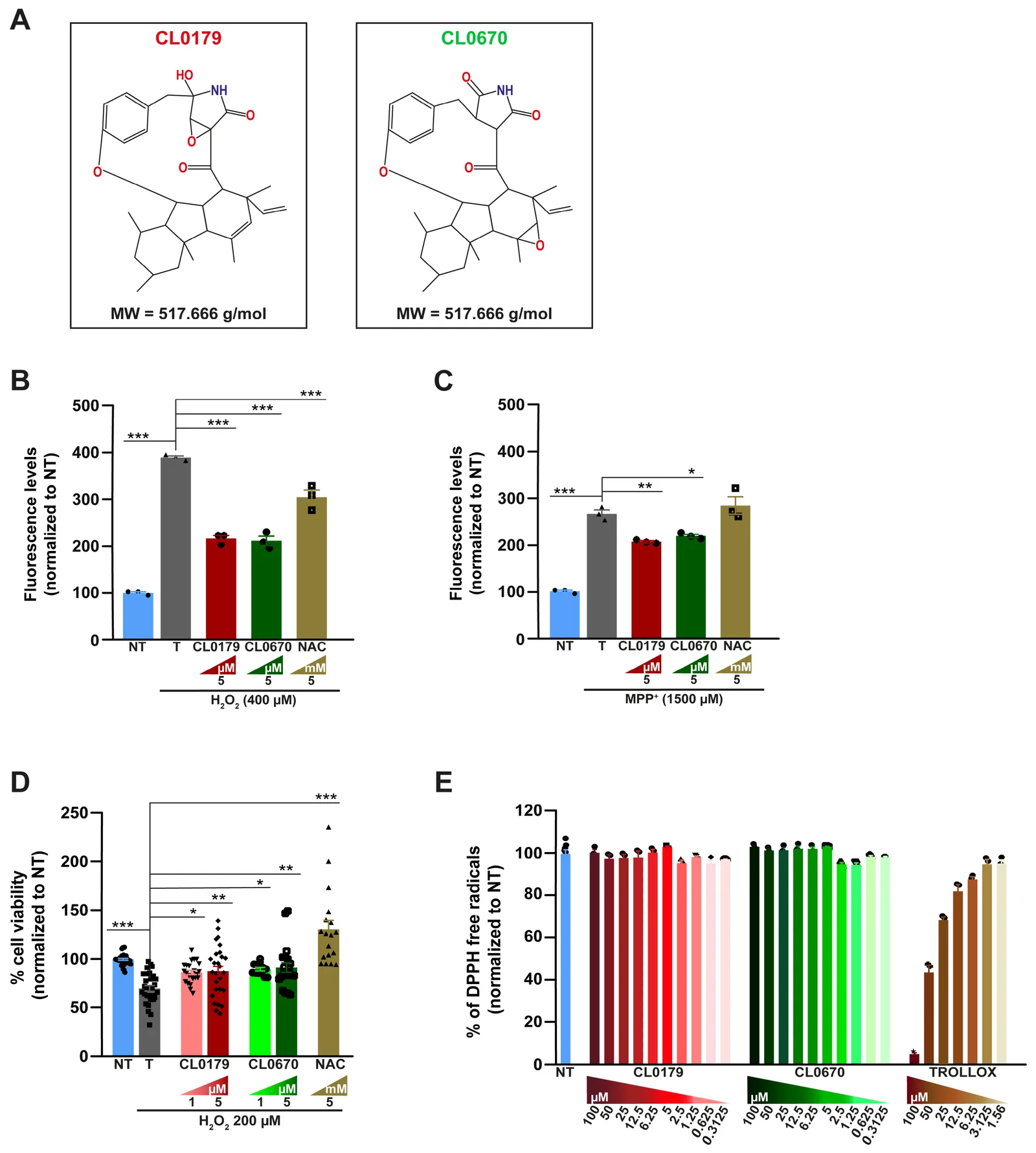
Structure, antioxidant-associated activity, and neuroprotective effects of the CL0179 analogue CL0670 in SH-SY5Y cells. (**A**) Chemical structure and molecular weight of CL0179 and CL0670. (**B**) ROS production in SH-SY5Y cells following H_2_O_2_ exposure. (**C**) ROS production in SH-SY5Y cells following MPP^+^ exposure. (**D**) Antioxidant-associated effect of CL0179 and CL0670 against H_2_O_2_-induced oxidative stress in SH-SY5Y cells. (**E**) Direct radical scavenging activity of CL0179 and CL0670. NT: untreated cells; NAC: N-Acetyl-L-Cysteine; T: H_2_O_2_ or MPP^+^ treatment; and ns: not significant. Data are expressed as mean ± SEM. * *p* < 0.05, ** *p* < 0.01 and *** *p* < 0.001 ((**B**,**D**) comparison to H_2_O_2_-treated cells; (**C**) comparison to MPP^+^-treated cells; (**E**) comparison to non-treated cells).

Despite its robust neuroprotective activity, the low production yield of CL0179 limited large-scale studies, prompting the development of a more accessible analogue with retained biological activity. This effort resulted in the semisynthetic derivative CL0670 (Figure 8A), obtained via epoxidation of a structurally related metabolite produced by the same fungal strain in substantially higher quantities. The pharmacological safety profile and pharmacological selectivity of CL0670 showed minimal effects across the ion channel panel with negligible activity (1.47%) of hERG inhibition, indicating low predicted cardiotoxic liability (Supplementary Figure S3A). Moreover, it displayed restricted pharmacological profiles against enzymes, kinases, monoamine transporters, and GPCR-related pathways (Supplementary Figure S3B) and a moderate, antagonist profile involving α-adrenergic, ETA, muscarinic, dopaminergic, serotonergic, and opioid receptors (Supplementary Figure S3C).

To determine whether the neuroprotective and antioxidant-associated activities of CL0179 were preserved in its analogue CL0670, intracellular ROS levels were measured in SH-SY5Y cells following H_2_O_2_ or MPP^+^ exposure. As expected, all insults markedly increased ROS levels, which were significantly attenuated by both alkaloids (Figure 8B,C) and improved cell viability following H_2_O_2_ exposure (Figure 8D). Together, these data demonstrate that CL0179 and its analogue attenuate oxidative stress, supporting their antioxidant-associated neuroprotective activity.

We next examined whether these effects could be explained by direct radical scavenging using a cell-free DPPH assay. Neither compound showed detectable DPPH radical scavenging activity over the tested concentration range, which encompasses the concentrations used in our cellular studies, whereas Trolox produced a marked concentration-dependent response, confirming assay responsiveness (Figure 8E). Thus, although CL0179 and CL0670 effectively reduce intracellular ROS at neuroprotective concentrations, their effects are unlikely to be primarily mediated by conventional direct radical scavenging.

**Figure 9:**
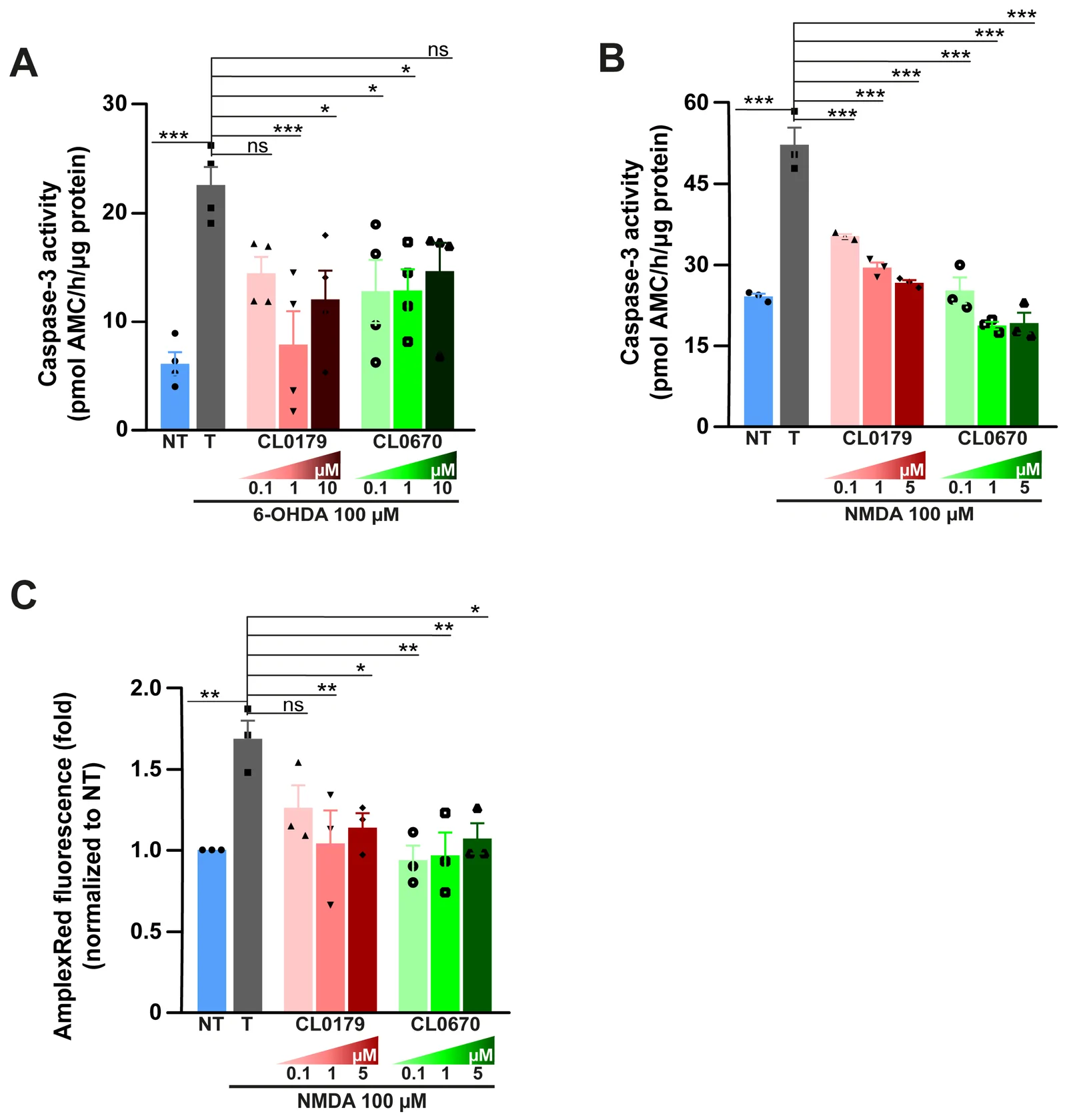
Neuroprotective and antioxidant associated effect of the CL0179 analogue CL0670 in primary mouse cortical neurons. (**A**) Neuroprotective effect of CL0179 and CL0670 against 6-OHDA (*n* = 3 independent experiments). (**B**) Neuroprotective effect of CL0179 and CL0670 against NMDA-induced damage (*n* = 3 independent experiments). (**C**) Antioxidant-associated effect of CL0179 and CL0670 against NMDA-induced damage in primary neuronal cultures. NT: untreated cells; T: 6-OHDA or NMDA treatment; and ns: not significant. Data are expressed as mean ± SEM. * *p* < 0.05, ** *p* < 0.01 and *** *p* < 0.001 ((**A**) comparison to 6-OHDA-treated cells; (**B**,**C**) comparison to NMDA-treated cells).

To assess whether the compound-mediated neuroprotection extended beyond toxin-induced oxidative and mitochondrial insults, we tested CL0179 and CL0670 in primary mouse cortical neurons exposed to NMDA. Unlike 6-OHDA, NMDA induces excitotoxicity through glutamate receptor overactivation and Ca^2+^ overload, leading to secondary mitochondrial dysfunction, thus providing an orthogonal model of neuronal injury [60]. Both compounds lacked toxicity across the tested concentration range (Supplementary Figure S2E,F). Notably, they significantly reduced 6-OHDA- and NMDA-induced apoptosis (caspase-3 activity; Figure 9A,B) and rapidly reversed NMDA-induced intracellular H_2_O_2_ accumulation (Figure 9C), demonstrating protection against both toxin-induced and excitotoxic neuronal injury.

Before advancing to in vivo studies the pharmacokinetic profiles of CL0179 and CL0670 were evaluated following intraperitoneal administration. Both compounds successfully crossed the BBB, reaching maximum brain concentration at 4 h post-injection, subsequently declining in a time-dependent manner (Supplementary Figure S4) with no adverse effects observed throughout the study.

**Figure 10:**
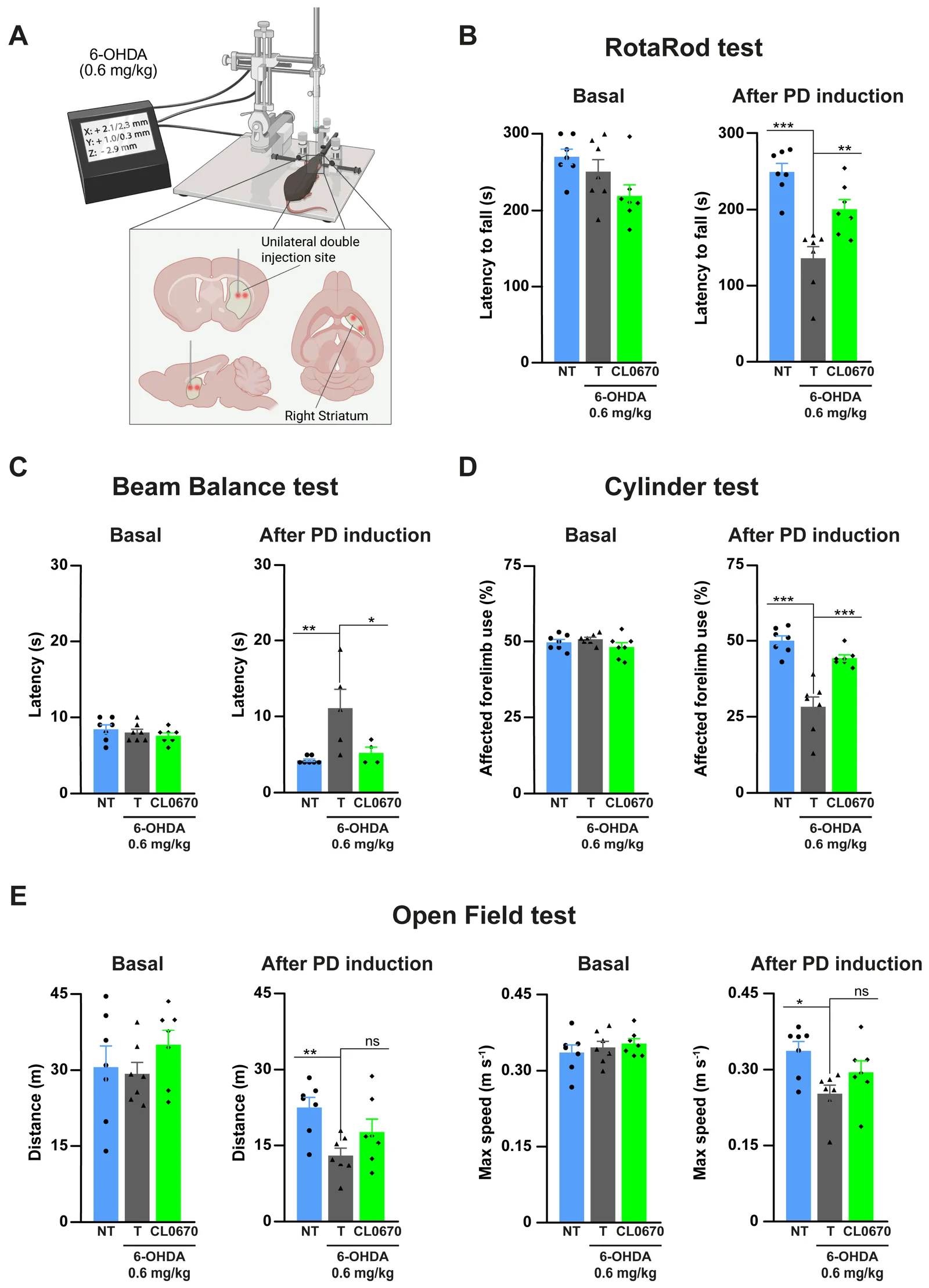
Effect of the CL0179 analogue CL0670 on the motor impairment induced by 6-OHDA in mice. (**A**) Schematic illustration of the 6-OHDA-induced Parkinson’s disease model. Mice underwent a double unilateral injection of 6-OHDA into the striatum to induce selective dopaminergic neurodegeneration. (**B**–**E**) Behavioral evaluation, before (basal) and 7 days after the Parkinson’s disease induction in the Rotarod test (**B**), beam balance test (**C**), cylinder test (**D**), and open field Test (**E**). Data are expressed as mean ± SEM (*n* = 7 animals per group). * *p* < 0.05, ** *p* < 0.01, *** *p* < 0.001 and ns: not significant ((**B**–**E**) comparison to 6-OHDA-treated cells).

To evaluate the translational potential of the compounds, we used the unilateral double intrastriatal 6-OHDA injection model (Figure 10A), which allows the assessment of PD symptoms including rest tremor, rigidity, postural instability, bradykinesia, and walking and gait difficulties [61]. Mice injected with 6-OHDA were treated with CL0670 or vehicle control, and a battery of behavioral tests that cover the spectrum of motor problems associated with dopamine deficits were performed. Under basal conditions, there were no differences in performance among the groups in all tests evaluated.

After PD induction, the animals treated with 6-OHDA alone fell earlier compared to controls when tested on the Rotarod test (Figure 10B and Supplementary Figure S5A), consistent with impaired motor coordination resulting from nigrostriatal dysfunction. Administration of CL0670 rescued this effect, although values did not fully return to control levels. While the animals treated with 6-OHDA alone required significantly longer to traverse the beam using the beam balance test (Figure 10C and Supplementary Figure S5B), animals treated with CL0670 partially reduced the latency to complete the task, indicating an improvement in balance performance and postural control.

To analize motor asymmetry, forelimb use was assessed using the cylinder test, analyzing the percentage of use of the affected limb, in this case the left paw, since we injected the 6-OHDA in the right striatum (Figure 10D and Supplementary Figure S5C). Animals treated with 6-OHDA alone used the affected limb half times less than controls, while administration of CL0670 partially, but significantly, restored forelimb use toward control values, demonstrating a substantial attenuation of the motor imbalance.

Finally, locomotor activity and bradykinesia-related parameters, alterations hallmarks of PD [62], were analyzed using the open field test by measuring the distance travelled and the maximum velocity reached by the animals (Figure 10E and Supplementary Figure S5D,E). Following 6-OHDA administration, mice exhibited a significant reduction in total distance traveled and maximum locomotor speed, consistent with impaired spontaneous movement and bradykinetic behavior. CL0670 treatment partially prevented these deficits, increasing both locomotor activity and maximal velocity relative to lesion-only animals.

Collectively, these findings demonstrate that CL0670 provides significant functional protection against 6-OHDA-induced motor impairment, with convergent improvements across multiple motor outcomes. While the limited availability of CL0179 precluded its evaluation in vivo, the efficacy of the structurally related analogue CL0670, together with the neuroprotective effects of CL0179 across multiple cellular models, supports the neuroprotective potential of this class of natural decahydrofluorene alkaloids and warrants further preclinical investigation.

## 4. Discussion

Despite decades of research, PD still lacks therapies capable of slowing or halting disease progression. Although natural bioactive compounds and mitochondria-targeted antioxidants have shown therapeutic promise, their clinical translation remains limited [17]. In this study, we identify fungal-derived decahydrofluorene alkaloids, as novel neuroprotective compounds that combine a favorable safety profile with robust activity across complementary cellular and animal models of PD. While the neuroprotective activity of CL0179 is completely new, the molecule was previously described as an antibiotic with activity against *Staphylococcus aureus* [63].

An important finding of our work is the restricted off-target pharmacological profile of CL0179. The compound exhibited minimal activity across ion channels, enzymes, transporters, and GPCRs, including negligible hERG inhibition, suggesting low cardiotoxic liability. Such selectivity is particularly attractive for neuroprotective drug development, where off-target activities frequently limit clinical progression. Together with the absence of intrinsic toxicity in multiple cellular systems, these results establish a favorable foundation for further therapeutic development.

Neurotoxin-induced SH-SY5Y models remain valuable tools in PD research, as they reproducibly recapitulate mitochondrial dysfunction, oxidative stress, and proteostatic impairment while providing scalable platforms for mechanistic studies and neuroprotective compound screening [39,60]. In this context, our data support a mitochondria-centered mechanism of action for CL0179. Across 6-OHDA-, rotenone-, and MPP^+^-based models, CL0179 attenuated mitochondrial depolarization, preserved mitochondrial network architecture, and improved cell survival. Importantly, the antioxidant-associated neuroprotective effects of CL0179 appear to extend beyond conventional direct radical scavenging. CL0179 reduced intracellular ROS following both H_2_O_2_ and MPP^+^ exposure while showing no detectable radical scavenging activity in the cell-free DPPH assay, supporting an indirect, cellular mechanism of protection against oxidative stress. Moreover, CL0179 increased SIRT1 activity under MPP^+^ stress, and pharmacological SIRT1 inhibition partially attenuated its ability to preserve mitochondrial membrane potential and cell viability. Together, these findings suggest that SIRT1 contributes to the stress-adaptive and mitochondria-preserving effects of CL0179 under oxidative and bioenergetic stress, although additional SIRT1-independent mechanisms are likely involved. This is particularly relevant given the central and potentially early role of mitochondrial dysfunction in PD pathogenesis.

Transcriptomic analyses further supported this interpretation. Rather than globally reversing the extensive transcriptional disruption induced by MPP^+^, CL0179 selectively restored a small set of genes involved in oxidative phosphorylation, respiratory chain activity, and mitochondrial bioenergetics. The limited number of DEGs observed after treatment argues against broad transcriptional rewiring and instead suggests a selective transcriptional response involving mitochondrial and stress-adaptation pathways. In agreement, the minimal transcriptional impact of CL0179 in healthy hDAn indicates limited perturbation of gene expression under basal conditions and is consistent with a more prominent transcriptional response under cellular stress. Such context-dependent activity, if confirmed, could represent an advantage over compounds that broadly perturb cellular homeostasis.

Another important aspect of this study is the validation of CL0179 in human disease-relevant models. While neurotoxin-induced SH-SY5Y models remain valuable screening tools, they incompletely capture the complexity of PD [42]. By contrast, patient-derived *LRRK2*-G2019S astrocytes and hiPSC-derived hDAn provide a more physiologically relevant context that incorporates human genetic background and cell-type-specific vulnerability. In *LRRK2*-G2019S astrocytes, CL0179 reduced ROS accumulation, α-synuclein buildup, and mitochondrial network abnormalities. Complementing this genetic glial model, CL0179 attenuated toxin-induced mitochondrial depolarization and preserved viability in hDAn, the primary neuronal population affected in PD. The convergence of these effects across neuronal and astroglial compartments suggests that the compound targets core pathogenic mechanisms shared among distinct PD-associated cellular contexts.

A common limitation of natural products is the difficulty of obtaining sufficient quantities for preclinical development. This challenge was also encountered with CL0179, whose low production yield restricted large-scale studies. To address this issue, we generated the semisynthetic analogue CL0670 from a structurally related fungal metabolite available in substantially higher amounts. Importantly, CL0670 retained neuroprotective activity, displayed a similarly favorable safety profile and successfully crossed the BBB. Moreover, the concomitant reduction in intracellular H_2_O_2_ accumulation and caspase-3 activation by both compounds in primary mouse cortical neurons indicates that these compounds attenuate oxidative and apoptotic responses associated with NMDA-induced excitotoxicity, potentially contributing to neuronal resilience against the calcium-dependent mitochondrial stress associated with excitotoxicity.

To determine whether the neuroprotective effects observed in cellular models translated into improvement of behavioral outcomes in vivo, we selected the unilateral intrastriatal 6-OHDA model, a robust proof-of-concept paradigm. In this model, we observed convergent improvements in motor coordination, balance, locomotor activity, and motor asymmetry in CL0670-treated mice, demonstrating a broad functional protective effect against 6-OHDA-induced motor impairment. However, certain limitations of this model have to be considered. Although intrastriatal 6-OHDA induces progressive retrograde degeneration of nigral dopaminergic neurons following initial striatal terminal damage [64,65], its temporal course is substantially faster than the slowly progressive neurodegeneration of human PD and does not reproduce key disease features such as α-synuclein aggregation, Lewy body pathology, or multisystem involvement. Therefore, while the beneficial effects of CL0670 provide proof-of-concept efficacy in an established model of oxidative and dopaminergic injury, validation in complementary chronic and α-synuclein-based models will be required.

Even though CL0179 could not be directly evaluated in vivo, the concordant neuroprotective effects observed across SH-SY5Y cells, primary neurons and human iPSC-derived dopaminergic neurons support the robustness of this compound family across complementary experimental systems and strengthen the translational relevance of this novel class of natural compounds.

Collectively, our findings identify a previously unrecognized class of fungal-derived compounds that promote mitochondrial resilience and preserve cellular homeostasis in cellular models of neurotoxic stress, while conferring functional protection in an in vivo model of dopaminergic injury. These results highlight decahydrofluorene alkaloids as promising candidates for the development of disease-modifying therapies targeting mitochondrial dysfunction in PD and potentially other neurodegenerative disorders characterized by bioenergetic failure.

## Data Availability

Data Availability Statements are available in https://www.ncbi.nlm.nih.gov/geo/query/acc.cgi?acc=GSE345242 (accessed on 3 September 2026).

## Supplementary Materials

The following supporting information can be downloaded at: https://www.mdpi.com/article/10.3390/antiox15091151/s1. Figure S1: CL0179 attenuates Rotenone-induced neurotoxicity in SH-SY5Y cells. (**A**) Neuroprotective effect of CL0179 against Rotenone-induced damage (*n* = 4 independent experiments). (**B**) Depolarization of the mitochondrial membrane potential under Rotenone-induced damage (*n* = 5 independent experiments). NT: untreated; NAC: N-Acetyl-L-Cysteine; T: Rotenone treatment. Data are expressed as mean ± SEM. ** *p* < 0.01 and *** *p* < 0.001 (A,B: comparison to Rotenone treated cells). Figure S2: CL0179 displays no intrinsic toxicity in human astrocytes, human induced pluripotent cells, human ventral-midbrain progenitors, human dopaminergic neurons or primary cortical neurons. Cytotoxicity of CL0179 in astrocytes (**A**), human induced pluripotent cells (**B**), human ventral-midbrain progenitors (**C**) human dopaminergic neurons (**D**) or primary cortical neurons (**E**). (**F**) Cytotoxicity of CL0670 in primary cortical neurons. NT: untreated; DMSO: dimethyl sulfoxide; hiPSC: human induced pluripotent cells; hDNs: human dopaminergic neurons. Data are expressed as mean ± SEM. *** *p* < 0.001 (B,C: comparison to DMSO treated cells; E,F: comparison to non-treated cells). Figure S3: Safety pharmacology profiling of the CL0179 analogue CL0670. (**A**) Ion channel activity using QPatch HT and IonFlux HT electrophysiological platforms. (**B**) Enzyme, kinase, monoamine transporter, and GPCR functional assays, associated with neuroactive and inflammatory pathways. (**C**) Cellular receptor functional assays measuring agonist and antagonist activities across a broad range of GPCRs involved in neurotransmission, inflammation, cardiovascular regulation, and neuromodulatory signaling pathways. Figure S4: In vivo Pharmacokinetics of CL0179 and CL0670. CL0179 and CL0670 concentration in plasma and brain samples analyzed after 0, 5 minutes, 15 minutes, 30 minutes, 1 hour, 4 hours, 10 hours, 24 hours, and 48 hours. The half-life (t1/2), clearance (CLp), mean residence time (MRT), volume of distribution (Vd) and area under the curve (AUC). Data are expressed as mean (*n* = 2 mice). Figure S5: Effect of the CL0179 analogue CL0670 on the motor impairment induced by 6-OHDA in mice. (**A**–**D**) Difference between the baseline and 7-days (raw delta) of the behavioural evaluation in the rotarod test (**A**), beam balance test (**B**), cylinder test (**C**) and open field test (**D**) (*n* = 7 animals per group). (**E**) Representative trackplot of the animal’s movement and the mean spatiotemporal heatmap of the mobility of each group of animals for the open field test. NT: untreated; T: 6-OHDA treatment. Data are expressed as mean ± SEM. * *p* < 0.05, ** *p* < 0.01*** *p* < 0.001 (A–D: comparison to 6-OHDA treated cells).

## Abbreviations

The following abbreviations are used in this manuscript:

6-OHDA	6-hydroxydopamine
MPP^+^	1-Methyl-4-phenylpyridinium-iodide
DCFH-DA	2′,7′-dichlorodihydrofluorescein diacetate
MTT	3-(4,5-Dimethyl-2-thiazolyl)-2,5-diphenyl-2H-tetrazolium bromide
AMC	7-amino-4-methylcoumarin
BBB	Blood–brain barrier
DMSO	Dimethyl sulfoxide
DMEM	Dulbecco’s Modified Eagle’s Medium
DPBS	Dulbecco’s Phosphate-Buffered Saline
hDAn	Human dopaminergic neurons
hiPSC	Human induced pluripotent stem cells
ΔΨm	Mitochondrial membrane potential
NAC	N-Acetyl-L-Cysteine
NMDA	N-methyl-D-aspartate
PFA	Paraformaldehyde
PD	Parkinson’s disease
PBS	Phosphate-Buffered Saline
ROS	Reactive oxygen species
CECT	Spanish Type Culture Collection
