# Supplementary material for: Fungal-Derived Decahydrofluorene Alkaloids Promote Mitochondrial Resilience and Neuroprotection in Cellular and Animal Models of Parkinson’s Disease
Source: Antioxidants (Basel). 2026 Sep 10;15(9):1151. doi: 10.3390/antiox15091151 (PMC13603763; doi:10.3390/antiox15091151)
Supplement: Supplementary file 1 [file antioxidants-15-01151-s001.zip › antioxidants-4422295-supplementary.pdf]

Supplementary Materials:

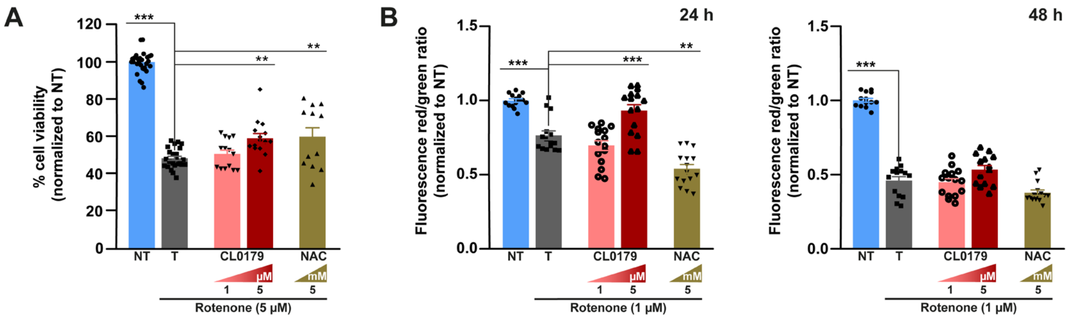

Figure S1. CL0179 attenuates Rotenone-induced neurotoxicity in SH-SY5Y cells. **(A)** Neuroprotective effect of CL0179 against Rotenone-induced damage (*n* = 4 independent experiments). **(B)** Depolarization of the mitochondrial membrane potential under Rotenone-induced damage (*n* = 5 independent experiments). NT: untreated; NAC: N-Acetyl-L-Cysteine; T: Rotenone treatment. Data are expressed as mean ± SEM. $^{**}P < 0.01$ and $^{***}P < 0.001$ (A,B: comparison to Rotenone treated cells).

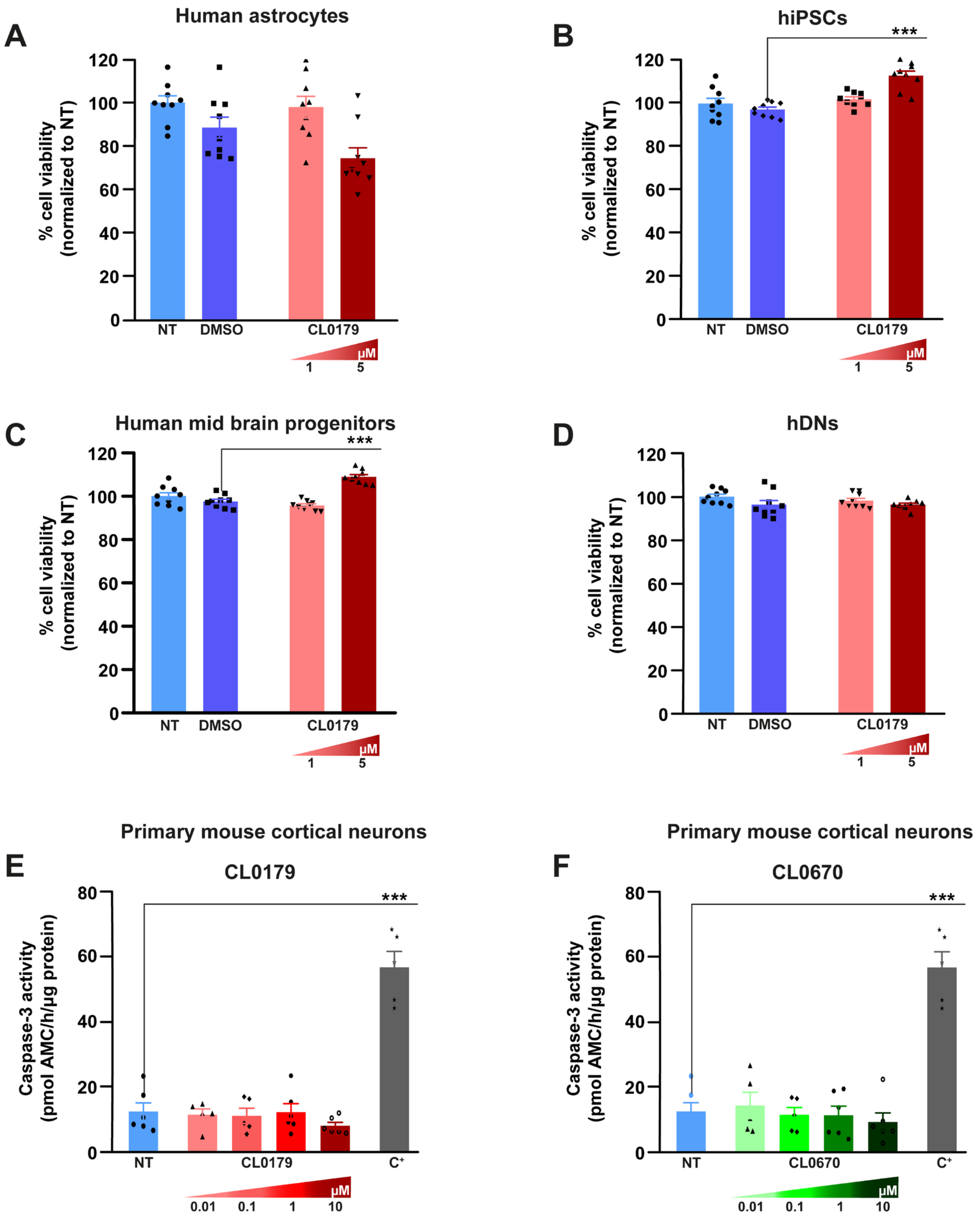

Figure S2. CL0179 displays no intrinsic toxicity in human astrocytes, human induced pluripotent cells, human ventral-midbrain progenitors, human dopaminergic neurons or primary cortical neurons. Cytotoxicity of CL0179 in astrocytes **(A)**, human induced pluripotent cells (**B**), human ventral-midbrain progenitors **(C)** human dopaminergic neurons **(D)** or primary cortical neurons **(E)**. **(F)** Cytotoxicity of CL0670 in primary cortical neurons. NT: untreated; DMSO: dimethyl sulfoxide; hiPSC: human induced pluripotent cells; hDNs: human dopaminergic neurons. Data are expressed as mean ± SEM. ****P* < 0.001 (B,C: comparison to DMSO treated cells; E,F: comparison to non-treated cells).

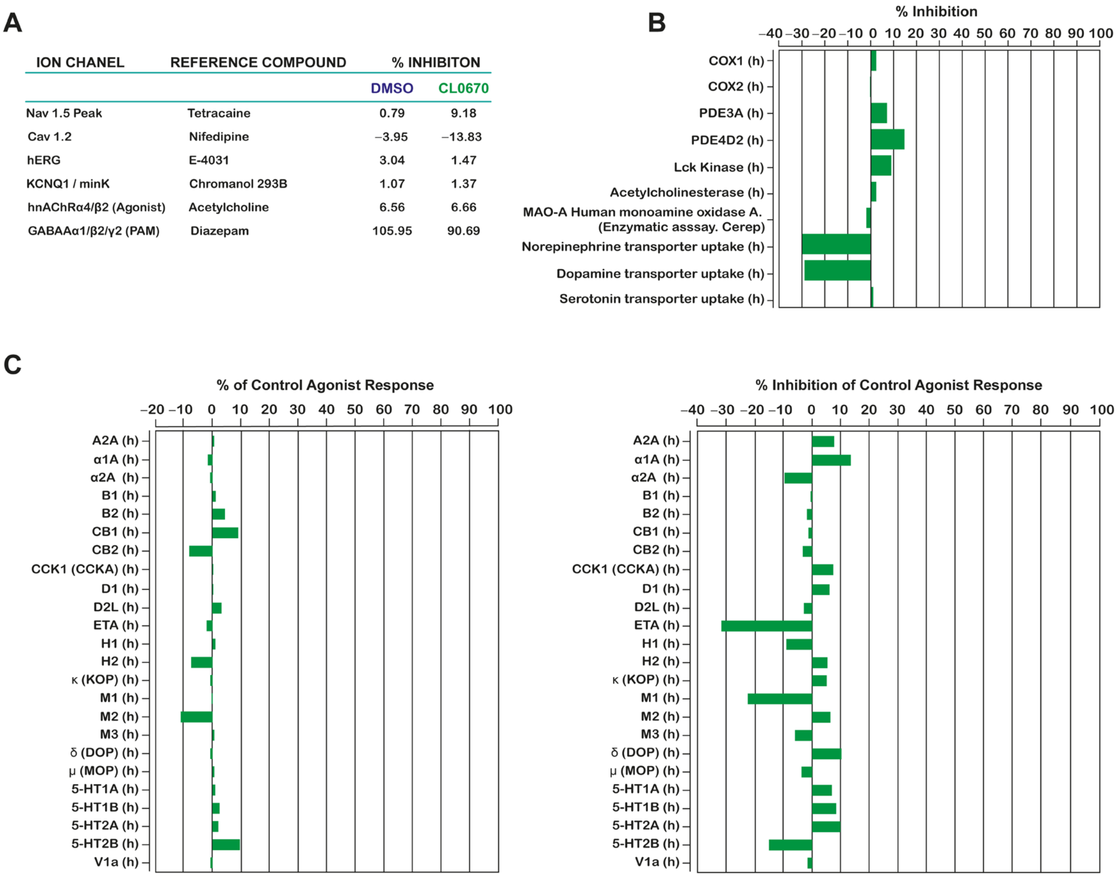

Figure S3. Safety pharmacology profiling of the CL0179 analogue CL0670. **(A)** Ion channel activity using QPatch HT and IonFlux HT electrophysiological platforms. **(B)** Enzyme, kinase, monoamine transporter, and GPCR functional assays, associated with neuroactive and inflammatory pathways. **(C)** Cellular receptor functional assays measuring agonist and antagonist activities across a broad range of GPCRs involved in neurotransmission, inflammation, cardiovascular regulation, and neuromodulatory signaling pathways.

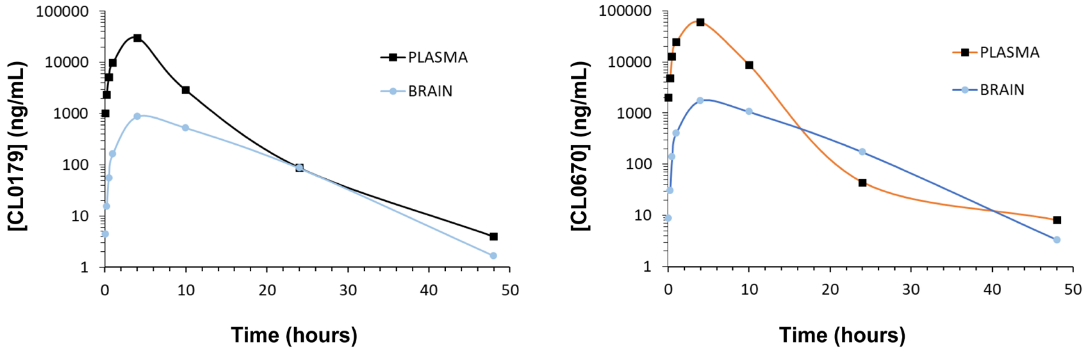

COMPOUNDS	SAMPLE	T 1/2	CLp	MRT	Vd	AUC (0-T)
CL0179 (150 mg/kg)	PLASMA	5.357	809.877	4.8	6.26	185200
	BRAIN	4.214	13130.247	9.6	79.846	11400
CL0670 (150 mg/kg)	PLASMA	7.546	1022.347	6.3	7.26	285200
	BRAIN	4.692	26170.212	11.2	93.656	21400

Figure S4. In vivo Pharmacokinetics of CL0179 and CL0670. CL0179 and CL0670 concentration in plasma and brain samples analyzed after 0, 5 minutes, 15 minutes, 30 minutes, 1 hour, 4 hours, 10 hours, 24 hours, and 48 hours. The half-life (t1/2), clearance (CLp), mean residence time (MRT), volume of distribution (Vd) and area under the curve (AUC). Data are expressed as mean (n = 2 mice).

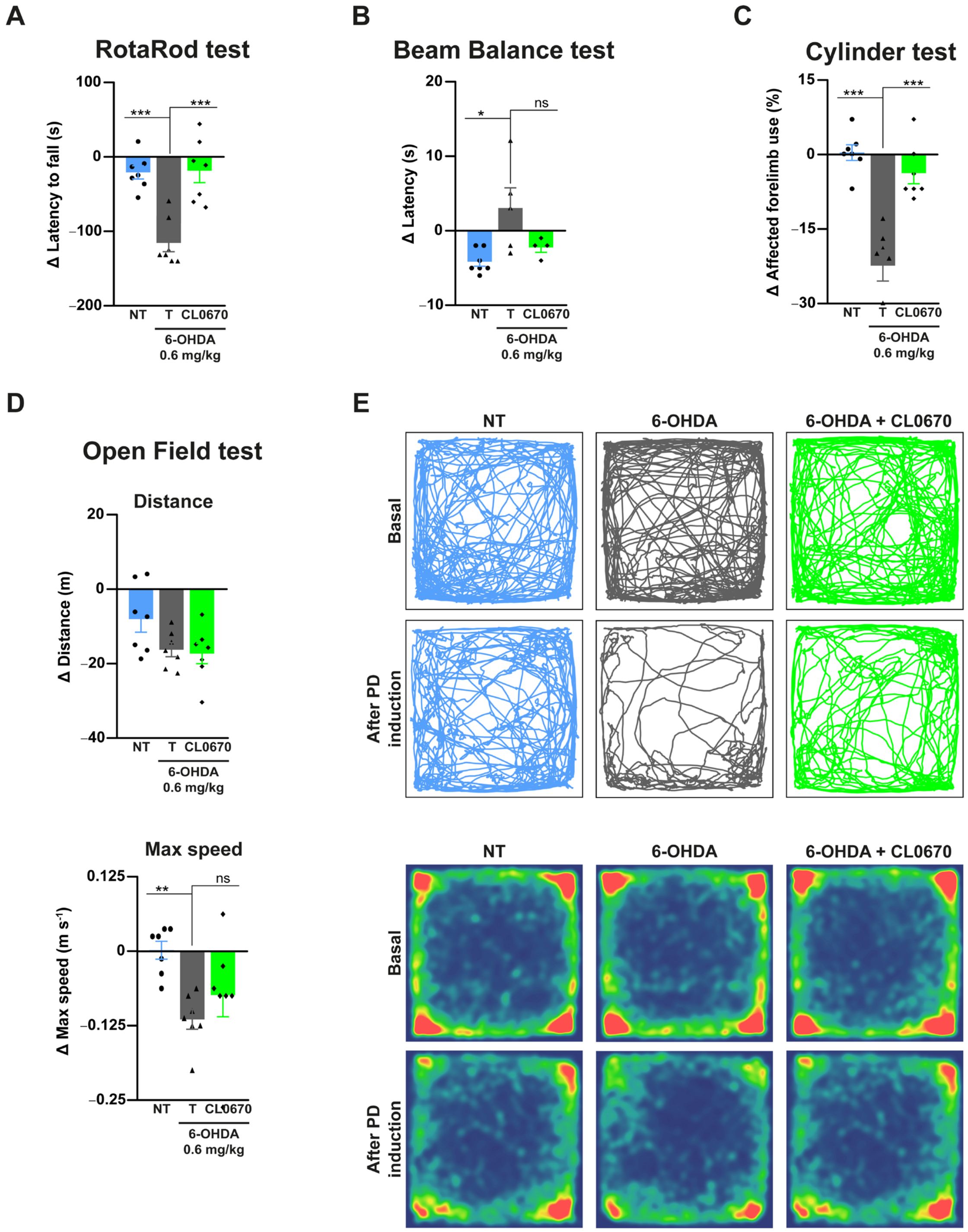

Figure S5. Effect of the CL0179 analogue CL0670 on the motor impairment induced by 6-OHDA in mice. **(A-D)** Difference between the baseline and 7 -days (raw delta) of the behavioural evaluation in the Rotarod test **(A)**, Beam Balance test **(B)**, Cylinder test **(C)** and Open Field Test **(D)** (n = 7 animals per group). **(E)** Representative trackplot of the animal's movement and the mean spatiotemporal heatmap of the mobility of each group of animals for the Open Field test. NT: untreated; T: 6-OHDA treatment. Data are expressed as mean ± SEM. *P < 0.05, **P < 0.01***P < 0.001 (A-D: comparison to 6-OHDA treated cells).

All Descripción del conjunto de datos is available at https://open.scayle.es/es/dataset/vazquez-jimenez-et-al-2026 (accessed on 3 September 2026).
